# Historical changes of the Mediterranean Sea ecosystem: modelling the role and impact of primary productivity and fisheries changes over time

**DOI:** 10.1038/srep44491

**Published:** 2017-03-14

**Authors:** Chiara Piroddi, Marta Coll, Camino Liquete, Diego Macias, Krista Greer, Joe Buszowski, Jeroen Steenbeek, Roberto Danovaro, Villy Christensen

**Affiliations:** 1 Institute of Marine Science (ICM-CSIC), Passeig Maritim de la Barceloneta, n° 39-45 08003 Barcelona, Spain; 2 European Commission, Joint Research Centre (JRC), Directorate D – Sustainable Resources, via Enrico Fermi 2749, I-21027 Ispra, Italy; 3 Ecopath International Initiative Research Association, Barcelona, Spain; 4 Institut de Recherche pour le Développement - UMR MARBEC (MARine Biodiverity Exploitation & Conservation) Avenue Jean Monnet, BP 171 34203 Sète Cedex, France; 5 Institute for the Oceans and Fisheries, University of British Columbia, Main Mall 2202, V6T1Z4 Vancouver, Canada; 6 Department of Life and Environmental Sciences, Università Politecnica delle Marche, 60131 Ancona, Italy; 7 Stazione Zoologica Anton Dohrn, 80121 Naples, Italy

## Abstract

The Mediterranean Sea has been defined “under siege” because of intense pressures from multiple human activities; yet there is still insufficient information on the cumulative impact of these stressors on the ecosystem and its resources. We evaluate how the historical (1950–2011) trends of various ecosystems groups/species have been impacted by changes in primary productivity (PP) combined with fishing pressure. We investigate the whole Mediterranean Sea using a food web modelling approach. Results indicate that both changes in PP and fishing pressure played an important role in driving species dynamics. Yet, PP was the strongest driver upon the Mediterranean Sea ecosystem. This highlights the importance of bottom-up processes in controlling the biological characteristics of the region. We observe a reduction in abundance of important fish species (~34%, including commercial and non-commercial) and top predators (~41%), and increases of the organisms at the bottom of the food web (~23%). Ecological indicators, such as community biomass, trophic levels, catch and diversity indicators, reflect such changes and show overall ecosystem degradation over time. Since climate change and fishing pressure are expected to intensify in the Mediterranean Sea, this study constitutes a baseline reference for stepping forward in assessing the future management of the basin.

Marine ecosystems around the world are increasingly pressured by a diversity of anthropogenic stressors, which include intensive fisheries and aquaculture, pollution, habitat loss and degradation, and species invasions^1^,^2^. These are acting synergistically with global climate change^3^. Since human stressors change over time^4^, the assessment of their temporal cumulative effects has been poorly studied and remains a challenging task^2^. Because these stressors are rapidly increasing, understanding how human interactions, the environment, and marine species interact and influence each other, and how such dynamics affect the sustainability of goods and services they provide, is of urgent importance. Currently this is a priority of many national and international regulations/initiatives (e.g., European Marine Strategy Framework Directive [MSFD; 2008/56/EC]; Convention of Biological Diversity, [CBD], Intergovernmental Platform on Biodiversity and Ecosystem Services [IPBES]) which promote the preservation of natural ecosystems and a sustainable use of biodiversity resources.

In support of these regulations, new comprehensive scientific tools have been developed with the goal of integrating the effects of the above-mentioned stressors into common frameworks in order to assist policy decisions^1^,^5^,^6^. Particularly in the context of ecosystem-based management approach (EBM), which assesses ecosystem dynamics rather than evaluating single resources and single threats, there has been a growing use of ecosystem models. These tools are improving their ability to predict complex system dynamics considering the impact of multiple pressures^7^ and assessing different policy objectives sought by management authorities^6^,^8^,^9^. Through hind-cast and forecast scenarios, ecosystem models allow for the quantitative assessment of the role of different stressors on ecosystem dynamics and for the calculation of model-based indicators able to evaluate whether an ecosystem and its services are maintained and used sustainably. Model-based indicators can complement data-based indicators^10^ and have been developed and widely used to capture the impact of specific pressures on marine ecosystems^11^,^12^, such as fishing, eutrophication. More recently, they have been utilized to assess socio-economic and governance issues^13^,^14^, as well as the cumulative impacts of multiple human activities^12^,^15^, informing management processes^9^,^16^.

This study applies the Ecopath with Ecosim (EwE) food web model approach to the Mediterranean Sea ecosystem as a whole, with the aim to evaluate temporal responses of species abundances and ecosystem dynamics to changes in primary productivity and fisheries (and their combination). The Mediterranean Sea is a highly diverse marine ecosystem that hosts 7–10% of the world’s marine biodiversity^17^,^18^. It is defined as “under siege” due to historical and current impacts of multiple stressors^19^. Among them, fishing practises, habitat loss and degradation, eutrophication, and more recently, the introduction of alien species and climate change effects^17^,^19^,^20^. Since the intensity of these stressors is increasing throughout most of the Mediterranean basin, temporal analyses are increasingly needed to inform effective current and future marine policies and management actions. In this study, we first quantify temporal dynamics of marine species in the Mediterranean Sea ecosystem as a whole, evaluating their historical dynamics. We then calculate a series of ecological indicators to analyse past ecosystem dynamics. Using the temporal-dynamic module of EwE, our specific goals are to investigate: 1) the temporal evolution of the Mediterranean marine ecosystem from 1950 to 2011 by developing a hind-cast scenario analysis that includes primary productivity, fisheries activities and food web dynamics; 2) differences and similarities in historical ecosystem dynamics using the primary productivity calculated by the EwE food web model and the primary productivity obtained from an hydrodynamic-biogeochemical model (GETM-MedERGOM); and 3) the structural and functional historical changes of the Mediterranean Sea ecosystem using specific model-based indicators.

In particular, the reason why we assessed temporal ecosystem dynamics using first the primary production from the high trophic level model, EwE, and then the primary production from the low trophic level model GETM-MedERGOM (n. 2 objective) was to evaluate if results from the modelling framework together (GETM-MedERGOM-EwE) were able to better capture the temporal evolution of the Mediterranean food web while accounting for dynamic forcing effects of climate and anthropogenic impacts (e.g., fishing)^11^. In recent years, under the growing need to provide guidance for biodiversity conservation and ecosystem based management, these so called end-to-end (E2E) models^5^,^21^ have been increasingly used to predict marine ecosystem changes and for scenario testing of climate change and anthropogenic impacts simultaneously^22^,^23^.

Thus, studies like the present are informative to support and implement European policies such as the Marine Strategy Framework Directive (MSFD; 2008/56/EC) that requires the development of strategies to achieve a “Good Environmental Status (GES)”. They can also support regional policies like the UNEP’s Mediterranean Action Plan (MAP) that aims at moving towards an ecosystem based management approach (EBM) for both EU and non-EU Mediterranean countries. The present study sets a baseline to further develop ecosystem analyses in order to facilitate the implementation of management policies and explore future plausible scenarios.

## Materials and Methods

### The baseline food web model of the Mediterranean Sea

**Figure 1:**
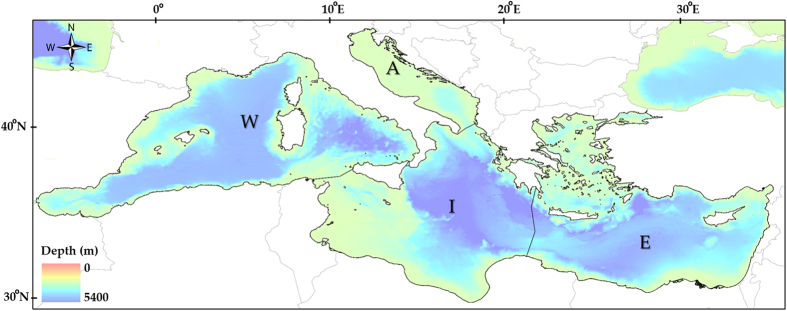
A representation of the Mediterranean Sea with the bathymetry and the four MSFD areas: Western Mediterranean Sea (W); Adriatic Sea (A); Ionian and Central Mediterranean Sea (I); Aegean and Levantine Sea (E). Map was originated using the GIS (Geographical Information System; ArcMap version 10.3; www.esri.com) software.

We used a previously developed food web model^24^ constructed with the Ecopath with Ecosim approach (EwE) using the Ecopath mass-balance module^25^ representing the whole Mediterranean ecosystem in the 1950 decade. We used the Ecopath model as a baseline to run temporal hind-cast (1950–2011) analyses, assessing the response of the Mediterranean marine ecosystem to changes in primary productivity and fishing effort. The baseline Ecopath model consisted of 103 functional groups, ranging from phytoplankton and invertebrates to top predator species and it was divided in four sub-models representing the four main areas of the Mediterranean basin: 1) Western Mediterranean Sea (W); 2) Adriatic Sea (A); 3) Ionian and Central Mediterranean Sea (I); and 4) Aegean Sea and Levantine Sea (E). These areas account for sub-regional differences in environmental and ecological characteristics of the ecosystem and are utilised also by the Marine Strategy Framework Directive (MSFD; Fig. 1).

**Figure 2:**
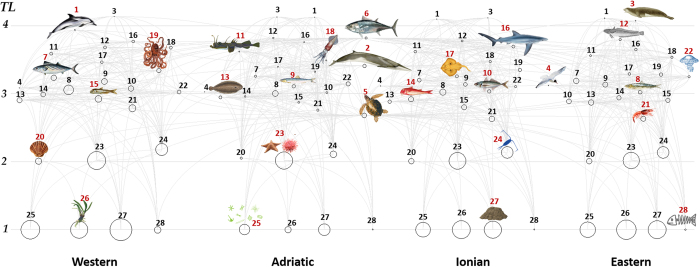
Flow diagram of the Mediterranean Sea ecosystem (year 1950 s) with the Western part being at the far left followed by the Adriatic, the Ionian and the Eastern. Each functional group is shown as a circle and its size is proportional to the log of its biomass. The functional groups are represented by their trophic levels (y-axis) and linked by predator-prey relationships showed as light grey lines. Numbers refer to functional group codes, which are reported in the legend, while those in red are graphically represented with a drawing. Numbers in the figure: 1. Piscivorous cetaceans; 2. Other cetaceans; 3. Pinnipeds; 4. Seabirds; 5. Sea turtles; 6. Large pelagics; 7. Medium pelagics; 8. European pilchard; 9. European anchovy; 10. Other small pelagics; 11. Large demersals; 12. European hake; 13. Medium demersals; 14. Small demersals; 15. Deep sea fish; 16. Sharks; 17. Rays and skates; 18. Benthopelagic cephalopods; 19. Benthic cephalopods; 20. Bivalves and gastropods; 21. Crustaceans; 22. Jellyfish; 23. Benthos; 24. Zooplankton; 25. Phytoplankton; 26. Seagrass; 27. Detritus; 28. Discards. Drawings for species/functional groups 1–3, 5–6 and 8 are by Massimo Demma - http://www.massimodemma.it/-.

The food web model had the following key input variables: biomass (B), production/biomass ratio (P/B), consumption/biomass ratio (Q/B), diet composition, and fisheries catches and discards. The main trophic structure of the Mediterranean Sea EwE model is shown in Fig. 2 and species and/or functional groups included in the model are listed in Table S1. A full description and sources of information of the input and output parameters of the baseline Ecopath model are available in Piroddi *et al*.^24^ and are presented in S2-S3 Tables in the Supporting Information.

A set of pre-balancing (PREBAL^26^) analyses are presented in Figure S4 with the purpose of showing the coherency of the basic input parameters with respect to general rules/principles of ecosystem ecology. In particular, these rules include: 1. biomass estimates by functional group in the model, which span 5–7 orders of magnitude when arranged against their trophic levels; 2. slope of biomass (on a log scale) by functional group, which declines by 5–10% across all the taxa when arranged against trophic levels; 3. vital rates (P/B; Q/B) across taxa/trophic levels which decline with increasing trophic level^26^,^27^.

The Ecopath model constructed in Piroddi *et al*.^24^ included seven types of fishing fleets: trawlers, dredges, mid water trawlers, purse seiners, longliners, artisanal and recreational activities. In this study, these fleets were adapted due to a lack of time series of data regarding the number of vessels and gross tonnage (GT) for some of the fleets, which are important for estimating historical fishing effort. In particular, while recreational fishery was retained from the previous model, main commercial fisheries were divided in: 1. trawlers (which included trawlers and dredges); 2. purse seiners; 3. longliners and 4. artisanal fisheries. This new fishing fleets configuration was created to follow the same structure as in Sacchi *et al*.^28^, the main source of information for temporal time series data of number of vessels and gross tonnage (GT) for the above-mentioned fleets for each Mediterranean country for the period 1990–2010. For Italy and Greece, we were able to get longer time series data using detailed reconstructions respectively from Piroddi *et al*.^24^ for the 1950–2010 period, from Stergiou *et al*.^29^ for 1964–1989, and Moutopoulos *et al*.^30^ for 1990–2010. To estimate an overall trend of number of fishing vessels for the 1950–2010 period, for those countries with missing years, we assumed same trends as observed by Greer^31^ who reported the number of fishing vessels for each country of the world for 1950–2010. GT was extrapolated, for the missing years, as the average ratio of GT in the observed time periods, while number of days spent fishing were kept as the ratio of days at sea observed respectively in Sacchi *et al*.^28^ for the majority of the countries, Piroddi *et al*.^24^ for Italy, and Moutopoulos *et al*.^30^ for Greece. For Spanish and Italian trawlers, we complemented our trends with data from EVOMED^32^, a European project that assessed the evolution and technological improvement of fishing capacity for the major countries of the Mediterranean Sea for the early 1900–2010 period.

Fishing effort (kW*days^−1^) was calculated as the product of the number of fishing vessels, kW per vessel (inferred from their GT), and the number of days spent fishing. To account for improvements in technology (e.g., mobile phone, GPS, sonar, radio) that were not captured by kW as a measure of effort (Figure S5), a conservative technological “creep factor” of 1% as observed by Damalas *et al*.^33^ and EVOMED^32^ was applied from 1980 to 1995 while for the remaining periods (1950–1979 and 1996–2010) a 0.5% and ~ 1.9% (this value varied with gear type; see S6 Table) were respectively used following the work of Pauly and Palomares^34^.

### Temporal dynamic modelling and model derived indicators

The dynamic module of the EwE software, Ecosim^25^,^35^, uses a set of differential equations to estimate biomass fluxes for each species and/or functional group of the ecosystem as follows:


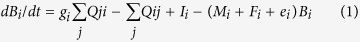


where dB_*i*_/dt is the biomass growth rate of group (i) during the interval dt, g_*i*_ is the net growth efficiency (production/consumption ratio), I_*i*_ is the immigration rate, M_*i*_ and F_*i*_ are natural and fishing mortality rates of group (i), e_*i*_ is emigration rate and B_*i*_ the biomass^25^. Calculations of consumption rates (Q_*ij*_) are based on the “foraging arena” theory^36^ where the biomass of prey i is divided between a vulnerable and a non-vulnerable fraction. This is represented as:


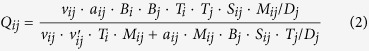


where v_*ij*_ and v’_*ij*_ is the vulnerability and expresses the rate with which prey move between being vulnerable and not vulnerable, respectively, a_*ij*_ is the effective search rate for i (prey) by j (predator), T_*i*_ and T_*j*_ are the relative feeding time for prey and predator, S_*ij*_ are the seasonal or long term forcing effects, M_*ij*_ are the mediation forcing effects and D_*j*_ are the effects of handling time as a limit to consumption rate. One important feature in Ecosim is the use of a vulnerability term for each interaction between a predator and a prey. Low values of vulnerability (close to 1) indicate that prey production determines the predation mortality (phenomenon also known as ‘bottom-up’ control) and that the predator is close to carrying capacity, while high values of vulnerability (e.g., 100) indicate that predator biomass determines how much prey is consumed (top-down control) and that predators are far away from carrying capacity^25^. Mixed effect (vulnerability = 2) is set as the default value in Ecosim.

The Ecosim approach was used here to fit the model to observed time-series of data using the sum of squares (SS) ratio between predicted and observed data as a metric for assessing model performance^37^. We used survey biomasses and catches for those functional groups with available information to compare predicted and observed data (S1). In particular, biomass time series for sea turtles, pinnipeds, benthic invertebrates and deep sea fish were taken from scientific literature, whereas for demersal species (functional groups n° 12–14; 16–19 and 21 in Fig. 2), European anchovy, European pilchard and large pelagic fishes, we used scientific surveys (e.g., MEDITS trawl survey and MEDIAS acoustic survey) and stock assessments data (S2). Catch data was taken from the United Nation’s Food and Agriculture Organization (FAO) database (FishStat: http://data.fao.org/database) available from 1950 to 2010. These time series were complemented with data (available per country) from the Sea Around Us Project (www.searoundus.org) to assign species to fishing fleet.

When applying the fitting procedure, we noticed that the baseline fishing mortality (Ecopath baseline in 1950 s) for the most commercially important target species (European pilchard, anchovy and hake) was relatively low (between 0.02 and 0.05) compared to the reference levels reported in the literature^38^,^39^,^40^. This initially caused a very low reaction of these species to changes in historical fishing effort and primary productivity. To correct these estimates and reflect a more appropriate fishing mortality for these three species, we used the reconstruction of the catches of the Sea Around Us Project and, in particular, for each country of the Mediterranean Sea, we considered the proportion of catch of these species relative to the total catch and applied it respectively in each of our sub-areas.

To fit the temporal dynamic model accounting for data quality/reliability in available time series, we weighted the time series using a factor either of 0.5 or 1 (0 indicating that time series are not considered in the calculation of SS and 1 indicating that they are fully considered^37^). For all catch time series and for European pilchards and anchovies in the Ionian and Eastern Mediterranean Seas, we used a weight of 0.5, while the rest of the time series were assigned a weight of 1. This was done to consider questionable catch statistics reporting (as identified in previous research studies^41^,^42^,^43^,^44^), and to consider poor data availability for forage fish in the Ionian and Eastern Mediterranean Sea (i.e., long time series of European anchovies and pilchards were available only for the Aegean^45^ and the Strait of Sicily^46^,^47^). The choice of using these weights (0.5 and 1, respectively) puts less/more emphasis on selected species/functional groups of the ecosystem; still, since there are different methods to determine weighting factors^27^, further work should be developed to assess the outcome of the fit procedure using alternative weights.

Fishing effort (Figure S5-1) and primary production (PP) anomaly (Figure S5-2a) over time were used as main forcing time series to drive the model. The PP anomaly results from an Ecosim automated procedure that searches for time-series relative values of annual production (expressed as P/B ratio) of producer groups (in our case phytoplankton and seagrass). This routine considers that if primary production changes over time then the total amount of energy that enters in the ecosystem changes, causing a cascading-up effect that increases or decreases food availability through the ecosystem^48^. Once estimated by Ecosim, the predicted relative PP anomaly was tested against the relative PP (from phytoplankton) time-series data (Figure S5-2b) obtained from a biogeochemical model GETM-MedERGOM^49^ for the same time period using the Spearman’s rank-order correlation test (suitable for non-parametric data). Seagrass productivity, since it was not assessed in the biogeochemical model^49^, was excluded from the correlation test.

In addition, we re-run the Ecosim model using the relative PP time-series data from the biogeochemical model to compare and assess the model fit and results using the two different PP time series data (relative PP anomaly from Ecosim, and relative PP data from the biogeochemical model). As for fishing effort, since our reconstruction was done up to 2010, but the majority of our biomass time series were available until 2011, we decided to keep fishing efforts observed in 2010 constant until 2011.

The fitting procedure consisted of seven general steps (Table 1) following the same approach as described and applied by Mackinson^50^. This method uses the Akaike Information criterion (AIC)^51^,^52^:


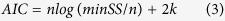


where n is number of observations, minSS is the minimum sum of squares resulting from the comparison of predicted with observed datasets, and k is the number of parameters, to test statistical hypotheses related to changes in predator-prey dynamics (also called vulnerabilities: Vs); changes in primary production (PP anomaly, considering the number of PP spline points (sPP) for smoothing the time series); impact of fishing and possible combinations of the above-mentioned factors (Table 1). The AIC is a tool used for model selection that penalizes for fitting too many parameters, and which is used to choose the “best” model (the one yielding the lowest AIC) considering a good fit and the least number of estimated parameters to do so. In this study, we used the second-order Akaike Information Criterion (AICc) calculated as follow:


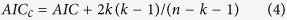


to account for small sample sizes (n of observations) in the dataset.

In our case, the fitting procedure was conducted five times: individually for the four sub-models (Western, Adriatic, Ionian and Eastern Mediterranean) as the majority of the functional groups are restricted to one sub-area only, and one extra time for the model representing the whole basin to fit highly migratory species (‘large pelagics’ and ‘sea turtles’ groups) that are allowed to move and feed in all four areas.

Once the temporal dynamic fitting procedure was completed, we used the “best” fitted models to calculate model-based indicators in each sub-area and for the whole Mediterranean Sea. To be able to compare these indicators with available ones from other regional seas, model-based indicators were selected from a list of indicators previously tested and assessed by international initiatives, mainly IndiSeas (“Indicators for the Seas”; www.indiseas.org; see e.g. refs 10,12). The list of selected indicators is presented in Table 2. Once estimated, we used the Spearman’s rank correlation to assess the significance and correlation between our suite of ecological indicators and time.

### Addressing uncertainty

The Monte Carlo routine built into EwE^25^ was applied in Ecosim to assess sensitivity of Ecosim’s output to the basic Ecopath input parameters (B, P/B, Q/B, EE), drawing input parameters from a normal distribution centered on the base Ecopath value and using a defined coefficient of variation, in this case set to 0.1^37^,^53^. Here, we run 1000 iterations, and the range of outputs (the 5^th^ and 95^th^ percentile) were plotted for both the fitted results (in our case time series of biomasses) and the model-based ecological indicators.

## Results

### Time series from the model fitting

The most statistically significant results in our model fitting exercise were obtained when trophic interactions, fishing and the primary productivity changes were included together in the model run (Step 7 in Table 3). Differences were found among the five areas with the “best” fitted models (lowest AICc) explaining from 50% to 69% of the variance of the data (Table 3). By looking at each area separately, the Ionian Sea sub-model was the one that showed the smallest improvement of prediction capabilities (thus the AICc estimates declined the least), while the Eastern followed by the Western Mediterranean were the areas with the highest improvement from the baseline AICc estimates. Both fishing and primary productivity drivers, when considered individually, were able to enhance the fit of all areas by ~16–~50% (when using the predicted PP anomaly) and by ~10–~37% when using fishing effort (steps 3 and 5 in Table 1).

The addition of trophic interactions to changes in PP anomaly alone (step 4 in Table 1) provided the second largest improvement for the Western, Ionian, and the whole Mediterranean Seas (AICc reduced further by ~10%). For the Adriatic Sea, this was obtained with the addition of trophic interactions to fishing effort (step 6 in Table 1). Also, different vulnerabilities were tested, and the largest enhancement was obtained using high vulnerabilities (step 7 in Table 3) for both the sub-models (maximum predator prey-interactions or Vs: #24) and the additional Mediterranean model as a whole (maximum predator prey-interactions or Vs: #2). Despite differences observed both at species/functional group and at sub-regional level, Ecosim results revealed that the Mediterranean Sea was mainly driven by bottom-up control interactions and low vulnerabilities were dominant (Western: 46%; Adriatic: 75%; Ionian: 50% and Eastern: 54%), followed by top-down controls interactions indicated by high vulnerabilities (Western: 42%; Adriatic: 21%; Ionian: 25% and Eastern: 33%). Mixed effect interactions represented a smaller proportion (vulnerabilities close to 2; Western: 13%; Adriatic: 4%; Ionian: 25% and Eastern: 13%) (S 14).

When we tested for correlation between the PP anomaly, resulting from the Ecosim fitting procedure, and the PP from the biogeochemical model, in all the areas these were positively and significantly correlated. Only the Adriatic Sea showed a significant negative correlation (Table 4 and Figure S7).

**Figure 3:**
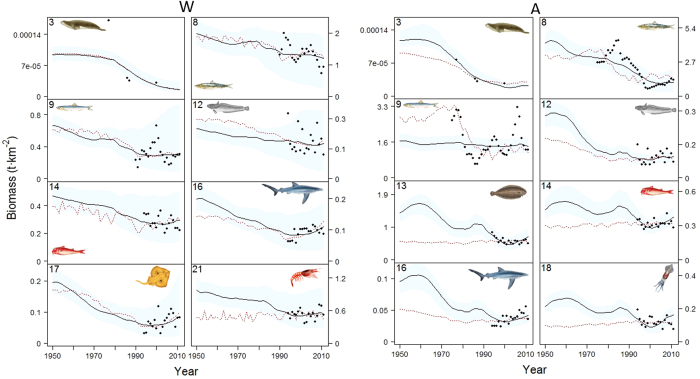
Representation of modelling fitting results for some functional groups occurring in the Western and Adriatic Seas for the period 1950–2011 (results for the rest of the groups are shown in Figure S8) Predicted biomass (t·km^−2^) is shown as solid black lines, while observed data is represented as black dots. Functional groups codes correspond to those given in Fig. 2. The predicted model (dashed red line) using modelled biogeochemical PP is also shown. Blue shadow represents the 95% percentile and 5% percentile obtained through the Monte Carlo routine. Drawings for species/functional groups 1–3, 5–6 and 8 are by Massimo Demma - http://www.massimodemma.it/-.

**Figure 4:**
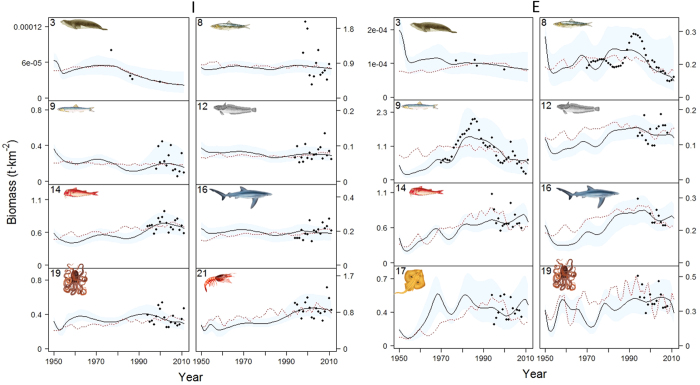
Representation of modelling fitting results for some functional groups occurring in the Ionian and Eastern Seas for the period 1950–2011 (results for the rest of the groups are shown in Figure S8). Predicted biomass (t·km^−2^) is shown as solid black lines, while observed data is represented as black dots. Functional groups codes correspond to those given in Fig. 2. The predicted model (dashed red line) using modelled biogeochemical PP is also shown. Blue shadow represents the 95% percentile and 5% percentile obtained through the Monte Carlo routine. Drawings for species/functional groups 1–3, 5–6 and 8 are by Massimo Demma - http://www.massimodemma.it/-.

Using the “best” fitted models, Ecosim reproduced satisfactorily the biomasses trends for some of the functional groups with available survey data in all sub-areas (Figs 3 and 4). Overall, forage fishes (functional groups n. 8–9), demersal fishes (n. 12–14) and invertebrates (n. 18–19 and 21) showed a good fit in the different sub-models, while deep sea fish (n. 15) and benthos (n. 23) were the least well fitted (Figs 3, 4 and S7). These latter groups are the ones with the fewest data points. A satisfactory fit was also shown for sharks and rays/skates groups (n. 16–17), and, despite only few observed records, also for pinnipeds (n. 3) (Figs 3, 4 and S8).

In the Western Mediterranean, based on the biomass trends by area, the predicted time series suggested a decreasing pattern for the biomasses of several functional groups (Figs 3 and S8). European pilchard (n.8) showed a decline from the beginning of our study period (1950), which became more pronounced in the last years of the observed time series. A similar result was also observed for medium (n. 13) and small (n. 14) demersal fishes, and pinnipeds (n. 3), although the model was not able to capture the sharp decline of these marine mammals in the 1970 s. As for sharks (n. 16), rays/skates (n. 17) the model confirmed a decrease in trends until the end of the 1990 s and a slight increase in the 2000 s decade. For European anchovy (n. 9) and hake (n. 12), Ecosim had difficulties reflecting observed variations in their biomass, although suggesting a decreasing trend for both species. A poor fit was observed for benthos (n. 23) and deep sea fish (n. 15), where only few data points were available. A good reproduction of biomass time series was found for crustaceans (n. 21) and benthopelagic cephalopods (n. 18) where the model was able to follow the majority of the fluctuations in time (Fig. 3 and S8). When the model was run using the PP from the biogeochemical model as an alternative primary productivity driver, we observed similar pattern (red dashed line in Figs 3 and 4) as the ones obtained using the PP Ecosim anomaly, and for certain species/functional groups (n. 8 and n. 21 in Fig. 3) the fit improved.

As for the Western Mediterranean, also in the Adriatic Sea, Ecosim suggested a decline for some top predators, demersal and pelagic fish and for some invertebrates (Figs 3 and S8). In particular, the model was able to capture the steep decline of pinnipeds (n. 3) observed in the area since mid-1970s^54^ and a less marked decrease of medium (n. 13) and small (n. 14) demersal fish observed in mid 1990s^55^,^56^. Ecosim captured some of the pattern observed for European hake (n. 12), sharks (n. 16), rays/skates (n. 17) suggesting a decline of the groups until the end of the 1990 s, followed by a slight increase or by fluctuations (in the case of European hake) in the last years of the studied period. An overall satisfactory match between predicted and available data was found for benthopelagic cephalopods (n. 18) where a decrease was captured since the beginning of the survey period, and for benthic cephalopods (n. 19) and crustacean (n. 21) where the model followed some of the fluctuation of the groups and a slight increase at the end of 2000 s. Again, the model did not represent the trends well for deep sea fish (n. 15; Figure S8). Regarding forage fish (n. 8–9), when we run the model using PP anomaly as driver, Ecosim was not able to reflect the decreasing biomass trend observed in European anchovies, while it was able to pick up a general decline for European pilchards. However, it was when we applied the PP from the biogeochemical model in the model run that Ecosim was able to follow the steep decline observed in European anchovies in mid 1970 s and improve also slightly the decline of European pilchard. For the other species/functional groups, different trends were observed using the two different PPs particularly in the decades before the beginning of our time series of observations (Figs 3 and S8).

The Ionian Sea resulted to be the area with less biomass changes during the years with available survey data (Figs 4 and S8). Except for pinnipeds (n. 3), where the model was able to pick up the decline since the late 1970, despite the presence of only few data points, all the other groups didn’t show any directional variation in time resulting mainly in a series of fluctuations. However, by looking at the overall time period (1950–2011), the model suggested a small increase in biomass since the beginning of 1990 s for small demersal fish and crustaceans. The model partly underestimated and was not able to capture the biomass trends for European pilchards and medium demersals (respectively n. 8 and n. 13; Figs 4 and S8), and it did not represent well the trend for benthos (n. 23; Figure S8). The use of PP from the biogeochemical model improved slightly the fit for crustaceans (n. 21), sharks (n. 16) and benthopelagic cephalopods (n. 18) while maintaining the same pattern observed with the PP anomaly.

**Figure 5:**
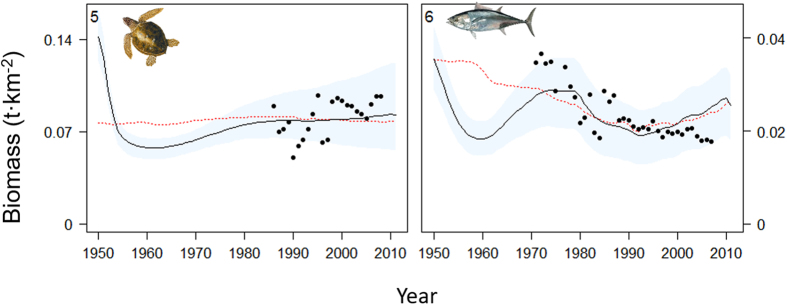
Representation of modelling fitting results for large pelagics and sea turtles in the Mediterranean Sea as whole for the period 1950–2011. Predicted biomass (t·km^−2^) is shown as solid black lines, while observed data is represented as black dots. Functional groups codes correspond to those given in Fig. 2. The predicted model (dashed red line) using modelled biogeochemical PP is also shown. Blue shadow represents the 95% percentile and 5% percentile obtained through the Monte Carlo routine. Drawings for species/functional groups 1–3, 5–6 and 8 are by Massimo Demma - http://www.massimodemma.it/-.

In the Eastern Mediterranean, different trends among species/functional groups were detected (Figs 4 and S8). Ecosim represented relatively well the biomass declines of European pilchards (n. 8) and anchovies (n. 9) since the 1990 s, despite underestimating the high peaks observed at the beginning of this decade. The model was able to capture the biomass trends for European hake (n. 12), small demersals (n. 14), sharks (n. 16), rays/skates (n. 17), benthic cephalopods (n. 19) and crustaceans (n. 21). All these groups showed similar patterns with signs of decrease in the 1990 s and fluctuations afterwards. An underestimation of biomass by the model was predicted for medium demersal fish (n. 13), deep sea fish (n. 15), benthopelagic cephalopods (n. 18), and benthos (n. 23) where the model was not able to reproduce observed trends and fluctuations (Figure S8). A good fit, even though for only few data points, was found for pinnipeds (n. 3) where the model was able to represent the fluctuation of these marine mammals over time (Fig. 4). The predicted trends obtained using PP from the biogeochemical model were similar to the ones found using the PP anomaly and for European hake (n. 12), small demersals (n. 14), sharks (n. 16), benthic cephalopods (n. 19) and crustaceans (n. 21) the fit slightly improved. Ecosim was able to represent the decrease in biomass of large pelagic fish (n. 6) particularly since the 80 s, while it failed to capture the fluctuation observed at the end of the 2000 s in the whole Mediterranean model for the two highly migratory species for which we had survey data: large pelagics and sea turtles. In the case of the sea turtles (n. 5), the model approximated the general increasing biomass trend, but it failed to reproduce its fluctuations over time (Fig. 5). We observed similar results with the PP from the biogeochemical model as a driver.

**Figure 6:**
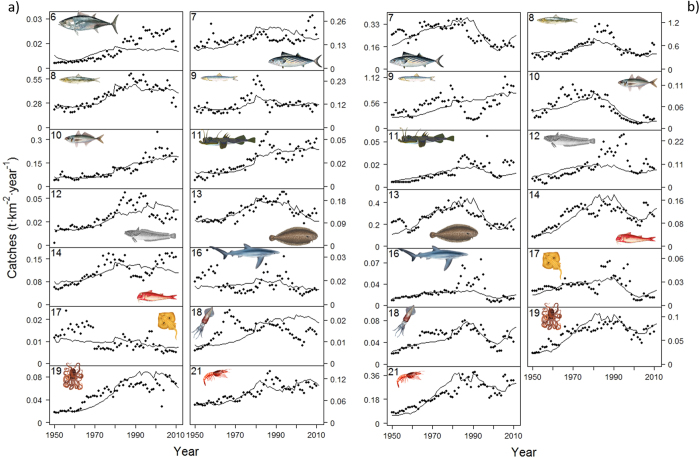
Predicted (solid lines) versus observed (dots) catches (t·km^−2^·year^−1^) for main commercially important functional groups of the Western Mediterranean (**a**) and Adriatic (**b**) ecosystems (1950–2011). Predictions obtained with the Mediterranean Sea model as whole for large pelagic catches are included in the Western Mediterranean plot (a. #6). Results for the Ionian and Aegean catches are shown in Figure S9. Drawings for species/functional groups 1–3, 5–6 and 8 are by Massimo Demma - http://www.massimodemma.it/-.

The time series of catch trends estimated for the five areas, when compared with independent data, showed a general satisfactory match (Figs 6 and S9): the sub-models overestimated or underestimated some fractions of the time series trends, but overall, they were able to capture long-term trends similar to those observed (Fig. 6). In the Western Mediterranean, an increase (up to the end of the 1990 s) and posterior decrease in catches were predicted for the majority of the groups with the exception of other small pelagic fish (n. 10), large demersal (n. 11), and benthic cephalopods (n. 19) that continued to increase even afterwards. Non-significant trend was simulated for rays/skates (n. 17), while the model was not able to reflect the trend observed for benthopelagic cephalopods (n. 18). Regarding large pelagic fishes (n. 6), catches predicted for the whole Mediterranean were similar to those observed until the 1980 s, but the predicted catches did not reflect the increase observed in the last two decades (Fig. 6).

In the Adriatic, as for the Western Mediterranean, the model simulated the decrease in catches observed in the beginning of the 1990 s for the majority of the functional groups while it did not succeed to pick up the sharp decline of European anchovies (n. 9) in mid 1970 s, and of European hake (n. 12) and sharks (n. 16) in the 1990 s (Fig. 6).

In the Ionian Sea, the model predicted the increase in catches until the end of 2000 s for the majority of the functional groups. For European hake (n. 12), medium demersals (n. 13), sharks (n. 16), rays/skates (n. 17) and benthic cephalopods (n. 19), though, such increase turned into a decrease approximately around the 1990 s (Figure S9). In the Eastern Mediterranean Sea, predicted results indicated the increase in catches for the majority of the functional groups until the 1990 s and the decline afterwards and they also captured the continuous increase for other small pelagic fishes (n. 10) and benthopelagic cephalopods (n. 18). On the other hand, simulated results did not match the sharp decline of sharks (n. 16) observed since the 1980 s in the region (Figure S9).

### Temporal model-based ecological indicators

**Figure 7:**
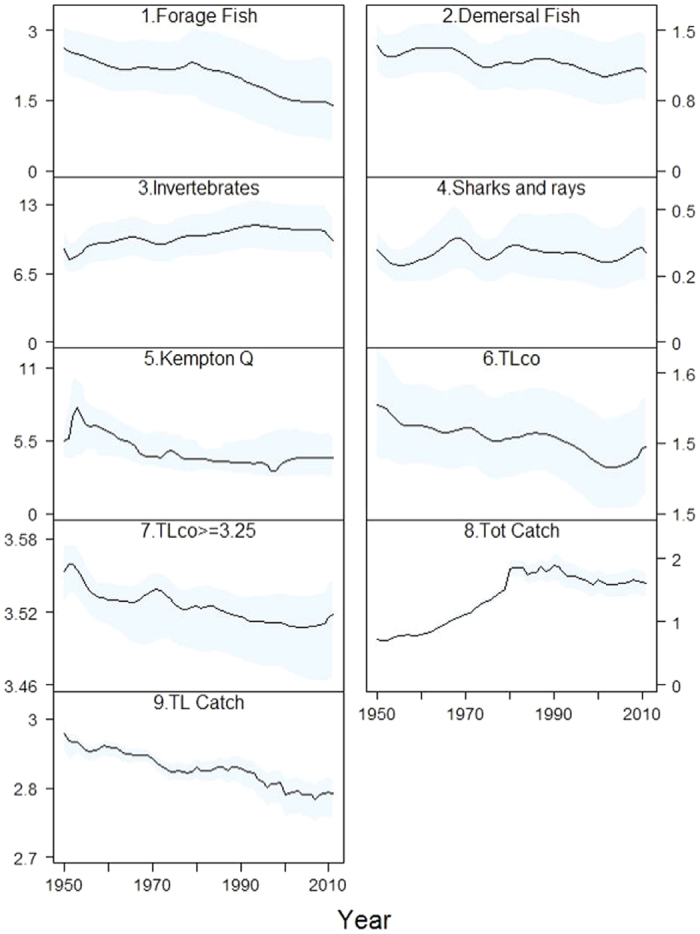
Ecological indicators 1. Forage fish biomass (t·km^−2^); 2. Demersal fish biomass (t·km^−2^); 3. Invertebrates biomass (t·km^−2^); 4. Sharks/rays and skate biomass (t·km^−2^); 5. Kempton Q: Kempton’s index of biodiversity; 6. TLco: Mean trophic level of the community; 7. TLco ≥ 3.25: Mean trophic levels of groups having trophic level ≥ 3.25 (excluding marine mammals, sea turtles and seabirds); 8. Tot Catch: Total catch (t· km^−2^ ·year-1); 9 TL Catch: Mean trophic level of the catches) estimated from the Ecosim results for the period 1950–2011 for the Catch Mediterranean Sea. Blue shadow represents the 95% percentile and 5% percentile obtained through the Monte Carlo routine.

**Figure 8:**
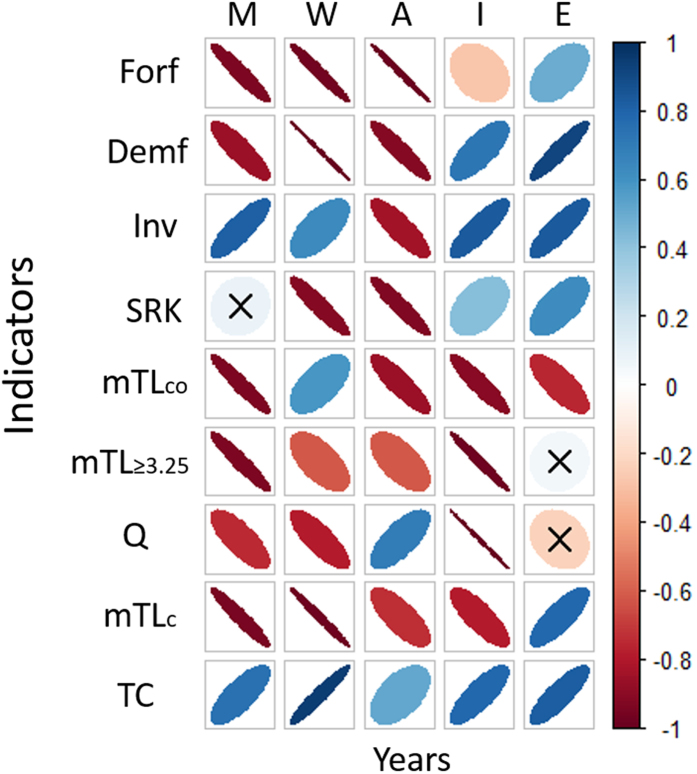
Spearman’s rank-order correlations representation between the suite of ecological indicators (Forf: Forage fish biomass (t·km^−2^); Demf: Demersal fish biomass (t·km^−2^); Inv: Invertebrates biomass (t·km^−2^); SRK: Sharks/rays and skate biomass (t·km^−2^); mTLco: Mean trophic level of the community; mTL ≥ 3.25: Mean trophic levels of groups having trophic level >3.25 (excluding marine mammals, sea turtles and seabirds); Q: Kempton’s index of biodiversity; mTLc: Mean trophic level of the catches; TC: Total catch (t· km^−2^ ·year^−1^) and time for the four sub-areas (Western: W; Adriatic: A; Ionian: I; Eastern and Levantine: E) and for the additional Mediterranean Sea as whole (Mediterranean: M). Positive correlations are displayed in blue and negative correlations in red color. Color intensity and the size of the eclipses are proportional to the correlation coefficients. At the right side of the graph, the legend color shows the correlation coefficients and the corresponding colors. The color follows a gradient according to the strength of the correlation. The width of the ellipses is related to correlation strengths with more diffused ellipses representing lower correlation strengths. When the indicators are non-significant (>0.05) are represented with an X symbol.

Trends in ecological indicators calculated from Ecosim temporal outputs showed different patterns if we looked at each sub-regional sea individually or at the Mediterranean ecosystem as a whole. For example, considering the entire Mediterranean Sea, a clear decreasing trend was observed in community biomass indicators like the forage fish biomass (on average −34%) and, to less extent, for demersal fish (−14%), the Kempton’s biodiversity index (−18%) and in all the trophic level indicators considered (TLco, TL ≥ 3.25 and TL Catch) (−5%) (Fig. 7). On the contrary, an increase was predicted for invertebrate biomass (+23%) while no clear trend was visible for sharks and rays/skates. Total catch was the only indicator that clearly increased in time (until 1990 s) (+189%) and that gradually decreased afterwards. These patterns were also reflected through the Spearman correlation test (Fig. 8).

Considering sub-regional seas (Figures S10–S13), we observed a clear decline of forage fish (−41%), demersal fish (−49%) and sharks/rays-skates (−60%) biomasses in the Western and Adriatic Seas, a fluctuation of these groups in the Ionian Sea while in the Eastern Mediterranean they respectively decreased, increased and fluctuated. Invertebrate biomass slightly decreased in the Adriatic Sea (−13%); fluctuated in the Western and Ionian Seas; and increased in the Eastern Mediterranean (+53%). The Kempton biodiversity index decreased in the Western (−49%) and in the Ionian Sea (−41%), it showed a slight increase in the Adriatic (+9%) while no clear trend was visible in the Eastern Mediterranean. Total catch increased in all the areas (+189%) until the beginning of 1990 s but in the Western and Ionian Seas started to fluctuate afterwards while in the Eastern and Adriatic Sea it gradually declined. As for the different trophic level indicators assessed, the mean TL of the community slightly increased in the Western Mediterranean (+10%) and decreased in the other sub-regions (−5%), while the mean TL ≥3.25 and mean TL catches decreased in all the seas (−4%) except in the Eastern Mediterranean where they respectively fluctuated with no clear trend and slightly increased (Figures S10–S13).

When we tested the significance and correlation of our suite of temporal ecological indicators, we noticed that in the Western and the Adriatic Seas the majority of the time series were negatively correlated with high significance (respectively 6 and 7 out of 9 indicators; Fig. 8). On the contrary, in the Ionian Sea and Eastern Mediterranean Sea, the community indicators (except for forage fishes in the Ionian that showed a weak negative correlation) were highly significant and positively correlated (Fig. 8). Also, we observed no significant and weakly correlated trends for mean TL ≥3.25 and Kempton biodiversity index in the Eastern Mediterranean Sea.

## Discussion

### Historical ecosystem drivers of the Mediterranean Sea ecosystem

Modelling results explained from 50% to 69% of the variability of historical time series data utilised in the present study. Both fishing pressure and PP anomaly played an important role in improving the model fit. In addition, our results indicated that the PP anomaly, representing the temporal variation of the primary productivity of the system, was the strongest driver upon the Mediterranean Sea ecosystem. This supports the results obtained from other studies^49^,^57^ that have shown how the Mediterranean Sea is driven by bottom-up processes where nutrient availability controls the biological characteristics of the region. The use of relative PP trends from a regional biogeochemical model helped validating our predicted PP anomaly trend and improved the temporal dynamics of selected species in the ecosystem (particularly for forage fish: European pilchards and anchovies). This was clearly visible, for example, in the Adriatic Sea where PP anomaly unsuccessfully reproduced the trends of European anchovies while PP trends from the biogeochemical model was able to better capture the dynamics. Since there are no official long-trend (from the 1950 s) records of primary production in the region (Macias *et al*. 2014), using a modelling framework that combines hydro-dynamic biogeochemical models with ecosystem models (such as EwE), becomes critically important, particularly in complex areas like the Adriatic Sea that show strong physical and biological oceanographic characteristics [decreasing gradient of trophic conditions from north to south^58^ ] and is also subjected to strong anthropogenic pressures (e.g., fishing)^59^,^60^. Currently there is a growing interest in these ensemble modelling frameworks^5^,^21^ in order to improve scientific capability to predict future ecosystem changes, and provide guidance for the setting of targets and implementation of management measures^8^,^61^. Accordingly to Macias *et al*.^62^, which explored potential changes in primary productivity in the Mediterranean Sea under different emission scenarios of future climate change, the Mediterranean basin will become warmer and more saline with consequences on the productivity of the region. The Western basin is expected to become more oligotrophic, associated to a surface density decrease influenced by the Atlantic waters, while the Eastern basin, on the contrary, is predicted to become more eutrophic due to a surface water density increase caused by increasing evaporation rate. Since these results are expected to alter the structural and functional processes shaping the Mediterranean food web, modelling tools like the one developed here are necessary in predicting the effects of climate variability and change on the future food web configurations.

Our study also highlights that fishing was an important driver affecting the dynamics of fish populations and invertebrates of the Mediterranean Sea ecosystem. This is in line with previous studies that documented the increasing impact of fishing in the Mediterranean Sea and the overexploitation of its marine resources^39^,^40^,^56^,^63^. Simulations, in fact, were able to reflect the impact of increased fishing effort in the basin starting, in all the four sub-areas, since the ‘50 s. Nominal fishing effort showed decreasing trends only after 2000; the only exception was found in the Eastern Mediterranean Sea where fishing effort showed a fluctuating trend in the 2000 s decade.

### Historical trends of biomass, catch and ecological indicators

We provide here new insights on temporal dynamics of major marine species/functional groups of the Mediterranean Sea ecosystem. In general, both biomass trends and ecological indicators revealed that the combined effect of excessive fishing pressure and changes in the primary productivity have altered the Mediterranean marine ecosystem over time, especially reducing the proportions of top predators and larger fish (e.g., pinnipeds, large pelagic fish) and increasing the abundance of groups at lower trophic levels (e.g., invertebrates). This was already observed from west to east in other studies, for example, in the Catalan^64^, Adriatic^55^,^59^ and Ionian^65^,^66^ Seas. Our results also show that forage fish species were observed to decrease, at a different time scale, in the majority of the studied Mediterranean sub-areas; with the only exception in the Ionian Sea where no clear trends were observed. These forage fish (mainly European pilchard and anchovy) constitute the bulk of fish catches in the Mediterranean Sea, accounting for almost 40% of total landings^67^ and they are of high commercial interest. Therefore, an increase of fishing mortality, together with changes in productivity, have affected these stocks throughout the Mediterranean Sea. As for the Ionian Sea, the results obtained here should be taken with caution. Our fitting analysis for the majority of the species/functional group in this area didn’t show any clear trend besides fluctuations over time. These results for the Ionian Sea disagree with several studies that have shown decreasing trend in the abundance of many commercial and uncommercial species in the area^47^,^65^,^68^. Poor model performance could be related to poor quality of the available data used in our study (e.g., for forage fish species, long time series were available only from Sicily), or to the fact that important additional factors were missing from our modelling analysis (e.g., changes in oceanographic and physical characteristics, quality of prey availability, etc.) that could be affecting Ionian Sea populations. This will need further research.

Trends in demersal fish stocks also show signs of decrease, both at regional and sub-regional scale (specifically in the Western and Adriatic Sea), while sharks (which in our model were mainly represented by demersal species, see S1) and rays/skates seemed to have declined in the Western and Adriatic regions, but not in the whole Mediterranean Sea. Part of these results are in line with historical^69^ and recent studies^39^,^40^,^70^, which pointed to increased fishing pressure and lack in gear selectivity as the reason why 85% of the assessed demersal stocks (including demersal sharks, rays and skates) are currently overexploited.

Overall, our estimates of species declines are conservative in comparison with results from fisheries stock assessments available for the Mediterranean Sea^39^,^40^,^71^. The reason behind is likely related to the lack of data particularly in the 1950s–1970s period, which probably contributed to lower the reduction captured by the model in term of abundance of certain functional groups. In addition, our model was built with functional groups, which in some cases aggregated a vast number of species including commercial and non-commercial ones, and/or considered the North and South Mediterranean Seas together. This is probably masking the largest declines of highly commercial species in the Northwestern and Central Mediterranean Sea areas that we observe when looking at these species individually.

A clear sign of change in the structure of the Mediterranean Sea ecosystem is visible from results of the mean trophic level of the community, mean TL ≥ 3.25 and Q diversity index (which includes those species or groups with TL ≥ 3) showing a decline since 1950 s and reflecting the decline of large predators/fish stocks and increase of lower trophic level organisms. These results are consistent with previous ecosystem assessments^17^,^63^, although it is important to bear in mind that these results were assessed considering both fitted and non-fitted groups. Caution should be taken when interpreting the results. Differences in ecological indicators (e.g., community and TL based indicators) were found among the different sub-regions analyzed and, because of lack of studies at sub-regional scale to validate these results, we would like to stress the need to further develop this work.

Regarding catches, the fitting procedure enabled us to detect issues related to landings data at the beginning of our survey period. Low fishing mortalities were observed in the ‘50 s, in each sub-area, for three very important commercial species (European pilchard, anchovy and hake). Mortality rates for these species were between 5 and 10 times lower than the average reference values reported for these fish stocks in the Mediterranean Sea^38^,^39^,^56^. This supports the hypothesis, already highlighted by several studies^30^,^41^,^72^,^73^, of poor quality of fisheries statistics, particularly in historical times (1950–1970). Part of this problem could be related to the different way fisheries data were collected and aggregated by the different countries and regional institutions^74^. Poor data quality and availability could also be explained by the presence of illegal, unreported and/or unregulated (IUU) fishing activities occurring in the region^41^,^42^,^43^,^44^ especially with regard to illegal nets and mesh sizes, the landing and marketing of undersized fish, and compliance with restrictions on fishing season and areas^39^. This highlights the need to utilize better catch data in modelling exercises in the Mediterranean Sea in order to account for more realistic fishing mortality estimates and trends, and guide proper management decisions. Recent catch reconstruction efforts, which aim at considering all types of fisheries removals (from reported and unreported landings to recreational landings and discards), have been constructed and are now available (www.seaaroundus.org) for the different countries of the Mediterranean Sea^75^. In this study, the catch reconstruction has been partly used to fill up gaps encountered in the FAO catch database (e.g., contribution of specific species/functional groups to fishing fleets and the proportion of the reconstructed catches of European pilchard/anchovy/hake relative to the total catch). Unfortunately, though, at the time this modelling exercise was completed, the reconstruction of total catches for Mediterranean countries was still being developed. Therefore, a necessary further step of this study should be the integration of such catch reconstruction in the input modelling parameters to compare results with the current implementation.

Despite limitations, our model was able to reflect the temporal trends of fisheries across the Mediterranean Sea, with a general increase in the total catch and a decline in the mean TL catch. Such patterns could reflect that catch composition, with a highly diversified targeted species, continues to change in time as a result of fisheries expansion to further and deeper fishing grounds^41^,^42^. A different picture emerges when looking at total catches per sub-regional area, where clear signs of decrease are noticeable mainly in the Adriatic and the Eastern Mediterranean Sea - and for the last simulation years also in the Western Mediterranean and Ionian Seas. These results are consistent with previous studies that have pointed out excessive fishing mortality and food web degradation caused by fishing in the Eastern and Adriatic fisheries^55^,^56^,^76^. On the other hand, the more stable catches observed in the Western Mediterranean and Ionian Sea could be the result of exploiting new species, as observed for the Mediterranean as a whole^42^.

The trophic level of the catches for the whole Mediterranean Sea and as well for the majority of the sub-areas (Western, Adriatic and Ionian Seas) presented a clear ‘fishing down’ effect^77^ that occurs when top predators and large sized fish are removed from the ecosystem and gradually replaced by lower trophic level organisms. Similar trends had been observed in the Mediterranean Sea, both at regional^77^, sub-regional^56^ and more local scale^10^,^78^. The only exception was found in the Eastern Mediterranean Sea where, contrary to the rest of areas, a situation of ‘fishing up’ was found. Accordingly to Stergiou and Tsikliras^79^, though, this might be a ‘false fishing up effect’ occurring when small pelagic fishes and invertebrates, with a low trophic level, and larger-size predators fish are both intensely fished and/or depleted.

### Management and conservation implications

The Mediterranean has been exploited for centuries, suffering the impacts of continuous and multiple anthropogenic pressures^19^,^80^. Because of increasing signs of deteriorations and degradations at species-, community- and ecosystem levels^19^,^39^,^80^, evidenced as well by this study, the basin is now of particular concern, and is a clear candidate for management actions to halt further decline and increase the sustainable use of marine resources^74^. Hindcasting analyses, as performed in this study, allow assessing historical changes in the ecosystem and in its marine resources, and are necessary pieces of the tool kit needed to support management and conservation processes.

Yet, to move towards better information to support regional policy and conservation plans, several additional steps should be developed in the near future.

First, spatial-temporal analyses able to identify spatial patterns that can directly assist spatial management actions (e.g., by prioritizing specific areas of concern), and facilitate the communication between scientists and policy makers, environmental managers, conservationists and the general public^80^ are needed to contribute to the recent Maritime Spatial Planning Directive (MSPD) of the European Commission^81^. A first attempt has been made in a recent study by Liquete *et al*.^82^, which assessed the delivery of five marine ecosystem services for the whole Mediterranean basin using several modelling approaches, both in time and space. However, as pointed out by these authors, more work is needed to be able to support management decisions.

Second, the integration of additional human stressors (e.g., aquaculture, invasive species, water warming, acidification, pollution, habitat degradation) as driving forces of species dynamics is needed to increase the reliability of this modelling exercise since marine ecosystems are impacted by simultaneous cumulative threats^19^,^80^. Currently the recent MSPD, which include the EU’s Blue Growth Strategy^83^ that supports sustainable growth in emergent marine sectors (e.g., aquaculture, coastal tourism, marine energies), is expected to impose further pressures on the Mediterranean Sea^19^,^84^.

Third, the development of forecast scenarios, including different future management actions, is crucial for the implementation of management plans. Future scenarios should follow the Intergovernmental Panel on Climate Change (IPCC) projections on climate-induced changes in sea surface temperature. They should also consider the relevant elements of the Common Fisheries Policy (CFP) on commercially important stocks to exploit them at Maximum Sustainable Yield (MSY) levels and the reduction of fishing effort needed to develop effective and appropriate policy and conservation plans in the region^39^,^56^.

In addition, this work emphasizes the need to use end-to-end modelling approaches as strategic tools for an explicit support to the decision-making process. Several end-to-end (E2E) models have now been developed^22^,^23^,^61^ to capture the feedbacks and multi-scale interactions that drive ecosystems dynamics with the goal of guiding responsible resource management decisions. As pointed out by Fulton^85^, there is no “one best E2E model”, on the contrary, a suite of tools should be considered given that such approaches are normally used to address questions at a scale where there is still a lot of uncertainty about how systems function. Our study advanced in this direction using results of complementary models together.

### Model assumptions and limitations

Modelling the Mediterranean Sea ecosystem is a challenging task, not only because of the complex dynamics that characterize this Large Marine Ecosystem (e.g., differences in environmental and biological features), but also because of the difficulties of gathering and integrating regional data^24^. Several gaps have been already identified and described in a previous work of Piroddi *et al*.^24^ which identified the lack of trophic information with a temporal dimension, the limited information on biomass of different species (especially of those non-commercially important species and deep-sea organisms), and the lack of reliable catch data and fishing effort. The absence of time series of seagrass productivity at regional scale is another constraint. Assessing the role and contribution of seagrass productivity on the overall marine primary productivity is critically important for understanding how changes in seagrass growth rates might impact the different compartments of an ecosystem^86^. In addition, the lack of historical data series (particularly between 1950 s and 1970 s), and the problems with data accessibility limit the potential resolution of modelling studies and the development of robust EBM approaches^24^,^74^,^87^. Therefore, more efforts should be dedicated to improve data collection and quality, and to make data better accessible for the region.

This study includes the best available regional data (see Supplementary Information) and highlights, when necessary, gaps and difficulties encountered in the modelling process (see below). To account for the uncertainty around the model parameters, we applied a Monte Carlo routine to evaluate model outputs sensitivity (in our case for biomasses and model derived indicators) to data uncertainty. Considering data uncertainty in model development is critical if the purpose of modelling is to inform policy/management processes^6^,^27^. Still, the majority of available modelling tools lack an approach to take uncertainty of modelled data (both input and output) into account^8^,^88^.

Although some time series were not always well replicated and uncertainty analyses can be improved as higher quality data becomes available, our modelling exercise reproduced several surveyed datasets in a satisfactory way and, as such, it is to date the best available representation of historical trends for the Mediterranean Sea as a whole, and a first step towards the integrated and historical understanding of this complex ecosystem.

## Conclusions

With anthropogenic pressures rapidly expanding in the Mediterranean Sea, there is a serious risk that these may push the system beyond the “point of no-return”, with consequence for marine biodiversity and the economies that depend on it, seriously constraining the ecosystem service options available to future generations. Ecosystem modelling tools can support the analysis and identification of the potential suitable options for ensuring the coexistence of sustainable human activities and the protection of healthy marine ecosystems. Temporal hind-cast analysis has enabled us to assess changes in the historical dynamics of species/functional groups inhabiting this system, quantifying the role and impact of changes in primary productivity and fishing pressure. Since climate variability and change in combination with fishing pressure is expected to intensify in the region, modelling approaches like the one presented here are necessary in predicting the effect of changes of the above-mentioned pressures on the marine food web. This constitutes an important first step further to advance in the regional assessment of the Mediterranean Sea ecosystem to inform conservation plans and management actions.

## Additional Information

**How to cite this article**: Piroddi, C. *et al*. Historical changes of the Mediterranean Sea ecosystem: modelling the role and impact of primary productivity and fisheries changes over time. *Sci. Rep.* **7**, 44491; doi: 10.1038/srep44491 (2017).

**Publisher's note:** Springer Nature remains neutral with regard to jurisdictional claims in published maps and institutional affiliations.

## Supplementary Material

Supplementary Information

## Figures and Tables

**Table 1:** Model fits following the seven steps proposed by Mackinson^50^, which include trophic interactions, fishery and environmental drivers (here changes in primary productivity).

#	Steps	Description
1	Baseline	Trophic interactions with default prey-predator vulnerabilities (vij = 2; mixed effect). No environmental or fishery data are used to drive the model.
2	Baseline and trophic interaction	Trophic interactions with different vulnerabilities. No environmental or fishery changes are used to drive the model.
3	Baseline and environment	The “PP anomaly” is used to drive the model. No fishery data are used to drive the model.
4	Baseline, trophic interactions and environment	No fishery data are used.
5	Fishery	Fishing effort is included as model driver. Trophic interactions are set as default and no environmental data are used.
6	Trophic interaction and fishery	No environmental data are used.
7	Trophic interactions, environment and fishery	All the components are jointly included in the model as drivers.

**Table 2:** Detailed description of modelled derived indicators with acronyms, definitions and references.

Ecological Indicator	Acronym	Definition and references
Community biomass	*Cm*	Index calculated at community level as the sum of the biomass only for those groups fitted to time series data (Unit: t/km^2^) 89. The Cm biomasses assessed were: Forage fish (European pilchard and European anchovy); Demersal fish (European hake, medium and small demersals); Sharks and rays (sharks, rays/skates); and Invertebrates (benthic and benthopelagic cephalopods, crustaceans and benthos).
Kempton Q species diversity index	*Qi*	Expresses biomass species diversity by considering those organisms with trophic levels 3 or higher^90^,^91^. The Kempton index is calculated as follow^91^: 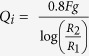 where Fg is the total number of functional groups in the model, R_1_ and R_2_ are the representative biomass values of the 10th and 90th percentiles in the cumulative abundance distribution.
Mean trophic level of community	*mTLco*	TL of the modelled community spans the whole ecosystem (living groups)^92^ including all functional groups (fitted and not fitted). TLs are calculated as follow^16^: 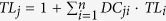 where j is the predator of prey i, DC_ji_ is the fraction of prey i in the diet of each predator j, and TL_i_ is the TL of prey i. The mean trophic level of community is calculated as follow^10^: 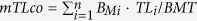 where B_MT_ is total biomass of the modeled ecosystem, B_Mi_ is the biomass of each species i in the model, and TL_i_ is the trophic level of species i as an output of the model (note: B_Mi_, DC_ji_ and TL_i_ vary in time).
Mean trophic level of groups with TL >3.25	mTL_3.25_	Calculated as the mTLco but including all functional groups (fitted and not fitted), excluding only marine mammals, seabirds and sea turtles mTL3.25^93^;
Total Catch	TC	Sum of all catches (Unit: t/km^2^/year)^94^.
Trophic level of the catch	TL_C_	TL of the catch for all retained species. Retained species are species caught in fishing operations, although not necessarily targeted by a fishery and which are retained because they are of commercial interest (i.e. not discarded)^92^. Trophic level of the catch is calculated as follow^10^: 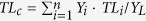 where Y_L_ is total landings, Y_i_ is the landing of species i and TL_i_ is the trophic level of species i (note: Y_L_, Y_i_ and TL_i_ vary in time).

**Table 3:** Results of the temporally dynamic fitting procedure of the Ecopath model from 1950 s to 2011 following the procedure suggested by Mackinson^50^ (Table 1).

Steps	Vs	sPP	min SS	k	AICc	%IF
**1. Baseline**						
*West*	0	0	191.0	0	−1768.0	
*Adriatic*	0	0	245.9	0	−1603.3	
*Ionian*	0	0	153.5	0	−1995.9	
*Eastern*	0	0	322.6	0	−1285.1	
*Med*	0	0	31.9	0	−227.9	
**2. Baseline and trophic interactions**						
*West*	1	0	190.9	1	−1766.7	−0.1
*Adriatic*	1	0	245.9	1	−1601.3	−0.1
*Ionian*	1	0	153.5	1	−1993.9	−0.1
*Eastern*	1	0	322.6	1	−1283.3	−0.2
*Med*	1	0	17.94	1	−226.1	−0.8
**3. Baseline and environment**						
*West*	0	6	144.1	6	−2049.7	15.9
*Adriatic*	0	28	156.1	28	−2037.2	27.1
*Ionian*	0	32	62.6	32	−2863.6	43.5
*Eastern*	0	28	167.2	28	−1931.4	50.3
*Med*	0	10	7.8	10	−306.5	34.5
**4. Baseline**, **trophic interactions and environment**						
*West*	23	3	103.0	26	−2357.7	33.4
*Adriatic*	23	13	136.7	36	−2164.0	34.9
*Ionian*	20	34	52.3	54	−3004.6	50.5
*Eastern*	22	29	137.8	51	−2089.1	62.6
*Med*	1	5	8.2	6	−308.9	35.6
**5. Fishery**						
*West*	0	0	160.8	0	−1946.9	10.1
*Adriatic*	0	0	172.7	0	−1985.7	23.8
*Ionian*	0	0	75.2	0	−2738.6	37.2
*Eastern*	0	0	211.6	0	−1736.6	35.1
*Med*	0	0	11.6	0	−280.2	22.9
**6. Trophic interactions and fishery**						
*West*	23	0	114.2	23	−2256.9	27.7
*Adriatic*	23	0	121.9	23	−2315.5	44.4
*Ionian*	23	0	62.9	23	−2876.7	44.1
*Eastern*	20	0	189.9	20	−1811.7	40.9
*Med*	2	0	9.5	2	−300.5	31.9
**7. Trophic interactions**, **environment and fishery**						
*West*	***22***	***5***	***60.1***	***27***	−***2917.2***	***65.0***
*Adriatic*	***23***	***6***	***104.5***	***29***	−***2469.2***	***54.0***
*Ionian*	***22***	***5***	***55.7***	***27***	−***2996.4***	***50.1***
*Eastern*	***21***	***12***	***133.1***	***33***	−***2165.0***	***68.5***
*Med*	***1***	***4***	***5.8***	***5***	−***353.8***	***55.3***

Vs is the number of vulnerabilities included in each iteration, sPP the number of primary production spline points (for smoothing of the time series) k is the number of parameters and %IF is the improved fit compared to the baseline AICc (#1). Vs and sPP are shown only for those models with the lowest Akaike Information Criterion (AICc). The “best” models (shown in bold and italics) are the ones yielding the lowest AICc and the one used to calculate model-based indicators

**Table 4:** Spearman’s rank-order correlations between the PP anomaly time series calculated by Ecosim and the PP from the biogeochemical model.

Sub-model	rho	p-value
West	0.37	7.5E-03
Adri	−0.59	6.5E-06
Ion	0.42	2.5E-03
East	0.49	3.0E-04
Med	0.82	2.2E-16

For a graphical representation of the correlation please refer to Figure S7 in the Supporting Information.
