# Supplementary material for: Historical changes of the Mediterranean Sea ecosystem: modelling the role and impact of primary productivity and fisheries changes over time
Source: Sci Rep. 2017 Mar 14;7:44491. doi: 10.1038/srep44491 (PMC5349533; doi:10.1038/srep44491)
Supplement: Supplementary Information [file srep44491-s1.pdf]

Supporting information

Historical changes of the Mediterranean Sea ecosystem: modelling the role and impact of primary productivity and fisheries changes over time

Chiara Piroddi[1,2*], Marta Coll[1,3,4], Camino Liquete[2], Diego Macias Moy[2], Krista Greer[5], Joe Buszowski[3], Jeroen Steenbeek[3], Roberto Danovaro [6,7], Villy Christensen[3,5]

[1]Institute of Marine Science (ICM-CSIC), Passeig Maritim de la Barceloneta, nº 39-45. 08003. Barcelona, Spain.

[2]European Commission, Joint Research Centre (JRC), Directorate D – Sustainable Resources, via Enrico Fermi 2749, I-21027 Ispra, Italy.

[3]Ecopath International Initiative Research Association, Barcelona, Spain.

[4]Institut de Recherche pour le Développement - UMR MARBEC (MARine Biodiverity Exploitation & Conservation) Avenue Jean Monnet, BP 171 34203 Sète Cedex, France.

[5]Institute for the Oceans and Fisheries, University of British Columbia, 2202 Main Mall, Vancouver B.C. V6T 1Z4.

[6]Department of Life and Environmental Sciences, Università Politecnica delle Marche, 60131 Ancona, Italy.

[7]Stazione Zoologica Anton Dohrn, 80121 Naples, Italy.

*Corresponding author

Email: cpiroddi@hotmail.com (CP)

S1. List of functional groups and fisheries and their abbreviations included in the Ecopath food web model and time series data sources used in the Ecosim dynamic modelling. Functional groups and fisheries categories are the same in each Marine Strategy Framework Directive (MSFD) area but their composition differ per area. This is not the case for highly migratory species (2. 'other cetaceans'; 5. 'sea turtles' and 6. 'large pelagic fish') that are common for all areas and they are allowed to move and feed in all the four sub-models [1]. NA, not available

#	Functional groups/fisheries	Source of biomass time series	Source of catch time series
1	**Piscivorous cetaceans (PC):** *Delphinus delphis, Stenella coeruleoalba, Tursiops truncatus*	NA	
2	**Others cetaceans (OC):** *Balaenoptera physalus, Globicephala melas, Grampus griseus, Physeter macrocephalus, Ziphius cavirostris*	NA	
3	**Pinnipeds (PI):** *Monachus monachus*	[2-13]	
4	**Seabirds (SB):** *Calonectris diomedea, Hydrobates pelagicus melitensis, Larus michahellis, Larus audouinii, Larus genei, Larus melanocephalus, Phalacrocorax carbo, Puffinus yelkouan, Puffinus mauretanicus, Sterna nilotica, Sterna sandvicensis, Sterna caspia, Sterna hirundo, Sterna albifrons, Sterna bengalensis*	NA	
5	**Sea turtles (ST):** *Caretta caretta, Chelonia mydas*	[14-26]	
6	**Large Pelagics (LP):** *Coryphaena hippurus, Tetrapturus belone, Thunnus alalunga, Thunnus thynnus, Xiphias gladius*	[27]	FishSTAT (FAO); ICCAT
7	**Medium pelagics (MP):** *Acanthocybium solandri, Alepes djedaba, Auxis rochei rochei, Auxis thazard thazard, Belone belone, Dicentrarchus punctatus, Euthynnus alletteratus, Katsuwonus pelamis, Lichia amia, Liza aurata, Orcynopsis unicolor, Pomatomus saltatrix, Sarda sarda, Scomber japonicus, Scomber scombrus, Scomberesox saurus saurus, Scomberomorus commerson, Seriola dumerili, Sphyraena sphyraena*	NA	FishSTAT (FAO)
8	**European pilchard (EP):** *Sardina pilchardus*	[28-44]	FishSTAT (FAO)
9	**European anchovy /EA):** *Engraulis encrasicolus*	[28,29,33-45]	FishSTAT (FAO)
10	**Other small pelagics (SP):** *Aphia minuta, Atherina hepsetus, Etrumeus sadina, Sardinella aurita, Spicara maena, Spicara smaris, Sprattus sprattus, Trachurus trachurus, Trachurus mediterraneus*	NA	FishSTAT (FAO)
11	**Large demersals (LD):** *Conger conger, Epinephelus aeneus, Epinephelus caninus, Epinephelus marginatus, Lophius piscatorius, Molva dypterygia, Muraena helena, Polyprion americanus*	International Bottom Trawl Survey in the Mediterranean (Medits database)	FishSTAT (FAO)
12	**European hake (HK):** *Merluccius merluccius*	International Bottom Trawl Survey in the Mediterranean (Medits database)	FishSTAT (FAO)

13	**Medium demersals (MD):** *Argyrosomus regius, Balistes capriscus, Campogramma glaycos, Cepola macrophthalma, Chelidonichthys lucerna, Chelon labrosus, Dactylopterus volitans, Dentex dentex, Dentex macrophthalmus, Dicentrarchus labrax, Epigonus telescopus, Eutrigla gurnardus, Labrus Merula, Lagocephalus sceleratus, Lepidopus caudatus,Lithognathus mormyrus, Lophius budegassa, Mugil cephalus, Naucrates ductor, Pagellus bogaraveo, Pagrus pagrus, Phycis blennoides, Platichthys flesus, Plectorhinchus mediterraneus, Sarpa salpa, Saurida undosquamis, Sciaena umbra, Scophthalmus maximus, Scophthalmus rhombus, Scorpaena scrofa, Solea solea, Sparisoma cretense, Sparus aurata, Spondyliosoma cantharus, Trisopterus luscus, Umbrina canariensis, Umbrina cirrosa, Zeus faber*	International Bottom Trawl Survey in the Mediterranean (Medits database)	FishSTAT (FAO)
14	**Small demersals (SD):** *Atherina boyeri, Boops boops, Chelidonichthys cuculus, Dicologlossa cuneata, Diplodus annularis, Diplodus sargus sargus, Diplodus vulgaris, Gobius niger, Helicolenus dactylopterus, Lepidorhombus whiffiagonis, Merlangius merlangus, Mullus barbatus barbatus, Mullus surmuletus, Nemipterus randalli, Oblada melanura, Pagellus acarne, Pagellus erythrinus, Phycis phycis, Scorpaena porcus, Serranus cabrilla, Serranus scriba, Synodus saurus, Trachinus draco, Trisopterus minutes, Uranoscopus scaber, Xyrichtys novacula*	International Bottom Trawl Survey in the Mediterranean (Medits database)	FishSTAT (FAO)
15	**Deep sea fish (DF):** *Alepocephalus rostratus, Argyropelecus hemigymnus, Bathypterois mediterraneus, Benthocometes robustus, Benthosema glaciale, Brama brama, Caelorhynchus caelorhynchus, Caelorhynchus mediterraneus, Cataetyx laticeps, Ceratoscopelus maderensis, Chalinura mediterranea, Chauliodus sloani, Chlorophthalmus agassizii, Coryphaenoides guentheri, Cyclothone braueri, Diaphus metopoclampus, Epigonus constanciae, Epigonus denticulatus, Epigonus telescopus, Halosaurus ovenii, Helicolenus dactylopterus, Hoplostethus mediterraneus, Hygophum benoiti, Hymenocephalus italicus, Lampanyctus crocodilus, Lepidion lepidion, Lepidopus caudatus, Lepidorhombus whiffiagonis, Micromesistius poutassou, Mora moro, Nettastoma melanorum, Nezumia aequalis, Nezumia sclerorhynchus, Notacanthus bonapartei, Notolepis rissoi, Paralepis speciosa, Polyacanthonotus rissoanus, Stomias boa, Trachyrhynchus trachyrhynchus, Trachyscorpia cristulata echinata*	[46]	
16	**Sharks (SK):** Alopias superciliosus, Alopias vulpinus, Carcharias taurus, Carcharodon carcharias, Centrophorus granulosus, Centrophorus granulosus, Centroscymnus coelolepis, Cetorhinus maximus, Chimaera monstrosa, Dalatias licha, Etmopterus spinax, Galeorhinus galeus, Galeus melastomus, Heptranchias perlo, Hexanchus griseus, Isurus oxyrinchus, Lamna nasus, Mustelus mustelus, Oxinotus centrina, Prionace glauca, Scyliorhinus canicula, Sharks nei, Somniosus rostratus, Squalus acanthias, Squalus blainville	International Bottom Trawl Survey in the Mediterranean (Medits database)	FishSTAT (FAO)

17	**Rays and skates (RS):** *Dasyatis pastinaca, Leucoraja naevus, Gymnura altavela, Mobula mobular, Myliobatis aquila, Rays and Skates nei, Raja asterias, Raja clavata, Raja montagui, Rhinobatos rhinobatos, Rostroraja alba*	International Bottom Trawl Survey in the Mediterranean (Medits database)	FishSTAT (FAO)
18	**Benthopelagic cephalopods (BPC):** *Alloteuthis media, Ancistroteuthis lichtensteini, Illex coindetii, Loligo vulgaris, Marine molluscs nei, Ostrea edulis, Sepia officinalis, Todarodes sagittatus*	International Bottom Trawl Survey in the Mediterranean (Medits database)	FishSTAT (FAO)
19	**Benthic cephalopods (BC)**: *Eledone cirrhosa, Eledone moschata, Marine molluscs nei, Octopus vulgaris*	International Bottom Trawl Survey in the Mediterranean (Medits database)	FishSTAT (FAO)
20	**Bivalves_gastropods (BG):** *Callista chione, Cerastoderma edule, Chamelea gallina, Crassostrea gigas, Donax vittatus, Littorina littorea, Marine molluscs nei, Mytilus galloprovincialis, Ostrea edulis, Pecten jacobaeus, Pecten maximus, Ruditapes decussatus, Venerupis pullastra, Venus verrucosa*	International Bottom Trawl Survey in the Mediterranean (Medits database)	FishSTAT (FAO)
21	**Crustaceans (CR):** *Aristaeomorpha foliacea, Aristeus antennatus, Carcinus aestuarii, Crangon crangon, Erugosquilla massavensis, Homarus gammarus, Maja squinado, Marine crustaceans nei, Marsupenaeus japonicus, Melicertus kerathurus, Metapenaeus monoceros, Nephrops norvegicus, Palaemon serratus, Palinurus elephas, Palinurus mauritanicus, Parapenaeus longirostris, Plesionika martia, Portunus pelagicus, Scyllarides latus, Squilla mantis*	International Bottom Trawl Survey in the Mediterranean (Medits database)	FishSTAT (FAO)
22	**Jellyfish (JF):** *Aequorea forskalea, Aurelia aurita, Pelagia noctiluca, Chrysaora hysoscella, Cotylorhiza tuberculata, Liriope tetraphylla, Mnemiopsis leidyi , Pleurobrachia rhodopis, Physalia physalis, Rhizostoma pulmo*	NA	
23	**Benthos (BE):** nematodes, copepods (and naupliar stages), polychaetes, oligochaetes, isopods, cumaceans, amphipods, acarians, ostracods, oligochaetes, tanaidaceans, cnidarians, kinorhynchs, turbellarians, gastrotriches, nemerteans, bivalves, priapulids (including larvae), cladocerans, decapods (larvae) and echinoderms	[46]	
24	**Zooplankton (ZO):** meso and macro zooplankton (amphipods, copepods, cladocerans, euphasids, mysids, pteropods)	NA	
25	**Phytoplankton (PH):** diatoms, dinoflagellates	NA	
26	**Seagrass (SE):** *Cymodocea nodosa, Posidonia oceanica, Zoostera marina, Zoostera noltii*	NA	
27	**Detritus (DE)**		
28	**Discards (DI)**		
29	**Trawlers (TR)**		
30	**Purse seiners (PS)**		
31	**Long liners (LL)**		
32	**Artisanal fisheries (AR)**		
33	**Recreational fisheries (RC)**		

S2. Input (light grey cells) and output (white cells) parameters of the Mediterranean marine ecosystem model for the 1950s period. Functional groups/species of the model are separated following the four Marine Strategy Framework Directive (MSFD) areas: Western (W); Adriatic (A); Ionian and Central Mediterranean (I); Aegean and Levantine (E).

#	Group name	Trophic level	Habitat area (fraction)	Biomass in habitat area (t/km²)	Biomass (t/km²)	Production / biomass (/year)	Consumption / biomass (/year)	Ecotrophic efficiency	Production / consumption
1	Piscivores cetaceans W	4.19	0.33	0.01	0.00	0.08	25.84	0.97	0.00
2	Others cetaceans	3.53	1.00	0.07	0.07	0.05	8.29	0.07	0.01
3	Pinnipeds W	4.20	0.33	0.00	0.00	0.08	13.15	0.90	0.01
4	Seabirds W	3.09	0.33	0.00	0.00	5.33	73.09	0.01	0.07
5	Sea turtles	2.68	1.00	0.02	0.02	0.19	2.78	0.14	0.07
6	Large Pelagics	3.94	1.00	0.44	0.44	0.35	2.50	0.04	0.14
7	Medium pelagics W	3.28	0.33	0.56	0.18	0.75	4.94	0.85	0.15
8	European pilchard W	3.13	0.33	0.55	0.18	0.99	8.45	1.00	0.12
9	European anchovy W	3.25	0.33	0.67	0.22	0.87	7.95	0.90	0.11
10	Other small pelagics W	3.14	0.33	0.36	0.12	0.75	6.63	0.90	0.11
11	Large demersals W	3.68	0.33	0.24	0.08	0.87	3.06	0.87	0.28
12	European hake W	3.81	0.33	0.28	0.09	0.60	2.80	0.91	0.21
13	Medium demersals W	2.94	0.33	0.79	0.26	0.70	6.40	0.92	0.11
14	Small demersals W	3.03	0.33	0.38	0.13	1.57	6.87	0.98	0.23
15	Deep sea fish W	2.97	0.33	0.85	0.28	0.70	3.50	0.99	0.20
16	Sharks W	3.85	0.33	0.36	0.12	0.42	3.48	0.10	0.12
17	Rays and skates W	3.34	0.33	0.28	0.09	0.80	3.67	0.83	0.22
18	Benthopelagic cephalopods W	3.69	0.33	0.32	0.11	2.00	9.00	0.96	0.22
19	Benthic cephalopods W	3.44	0.33	0.56	0.18	2.10	7.00	0.86	0.30
20	Bivalves_gastropods W	2.01	0.33	1.00	0.33	1.30	5.00	0.94	0.26
21	Crustaceans W	2.79	0.33	0.99	0.33	3.50	12.00	0.97	0.29
22	Jellyfish W	3.08	0.33	0.33	0.11	13.87	50.48	0.42	0.27
23	Benthos W	2.02	0.33	16.22	5.39	2.50	9.04	0.33	0.28
24	Zooplankton W	2.25	0.33	7.76	2.58	30.60	102.00	0.81	0.30
25	Phytoplankton W	1.00	0.33	18.40	6.11	197.00	--	0.18	--
26	Seagrass W	1.00	0.33	16.70	5.55	5.94	--	0.16	--
27	Piscivores cetaceans A	4.16	0.05	0.00	0.00	0.08	25.84	0.90	0.00
28	Pinnipeds A	4.19	0.05	0.00	0.00	0.08	13.15	0.55	0.01
29	Seabirds A	3.03	0.05	0.00	0.00	4.61	69.34	0.16	0.07
30	Medium Pelagics A	3.26	0.05	0.88	0.05	0.92	6.76	0.89	0.14
31	European pilchard A	3.00	0.05	4.32	0.23	0.80	9.19	0.31	0.09
32	European anchovy A	3.11	0.05	2.60	0.14	0.85	11.02	0.75	0.08
33	Other small pelagics A	3.02	0.05	0.53	0.03	1.00	11.29	0.48	0.09

34	Large demersals A	3.63	0.05	0.20	0.01	0.90	5.14	0.72	0.18
35	European hake A	3.86	0.05	0.28	0.01	0.40	1.85	0.79	0.22
36	Medium demersals A	2.96	0.05	0.39	0.02	1.10	5.57	0.99	0.20
37	Small demersals A	2.96	0.05	0.32	0.02	1.50	8.02	0.97	0.19
38	Deep sea fish A	2.88	0.05	0.61	0.03	0.70	3.50	0.98	0.20
39	Sharks A	3.79	0.05	0.10	0.01	0.50	4.00	0.26	0.13
40	Rays and skates A	3.41	0.05	0.12	0.01	0.64	4.10	0.77	0.16
41	Benthopelagic cephalopods A	3.58	0.05	0.22	0.01	2.70	9.00	0.88	0.30
42	Benthic cephalopods A	3.45	0.05	0.33	0.02	2.10	7.00	0.85	0.30
43	Bivalves_gastropods A	2.05	0.05	0.95	0.05	1.30	5.00	0.99	0.26
44	Crustaceans A	2.76	0.05	0.80	0.04	3.50	12.00	0.99	0.29
45	Jellyfish A	3.14	0.05	2.27	0.12	14.60	50.48	0.94	0.29
46	Benthos A	2.02	0.05	68.24	3.64	1.31	6.71	0.18	0.20
47	Zooplankton A	2.11	0.05	5.79	0.31	37.85	126.17	0.97	0.30
48	Phytoplankton A	1.00	0.05	15.00	0.80	140.00	--	0.33	--
49	Seagrass A	1.00	0.05	2.68	0.14	4.02	--	0.50	--
50	Piscivores cetaceans I	4.13	0.30	0.00	0.00	0.08	25.84	0.77	0.00
51	Pinnipeds I	4.16	0.30	0.00	0.00	0.08	13.15	0.35	0.01
52	Seabirds I	3.11	0.30	0.00	0.00	4.60	105.43	0.07	0.04
53	Medium Pelagics I	3.20	0.30	0.38	0.11	0.70	7.70	0.96	0.09
54	European pilchard I	3.02	0.30	0.48	0.14	0.94	8.68	0.97	0.11
55	European anchovy I	3.14	0.30	0.53	0.16	0.91	12.30	0.86	0.07
56	Other small pelagics I	3.04	0.30	0.28	0.08	0.86	8.36	0.95	0.10
57	Large demersals I	3.66	0.30	0.20	0.06	0.65	2.85	0.93	0.23
58	European hake I	3.86	0.30	0.13	0.04	0.65	3.40	0.96	0.19
59	Medium demersals I	2.89	0.30	0.65	0.20	0.90	8.13	0.82	0.11
60	Small demersals I	2.93	0.30	0.34	0.10	1.10	6.38	0.98	0.17
61	Deep sea fish I	2.80	0.30	0.59	0.18	0.70	3.50	0.81	0.20
62	Sharks I	3.72	0.30	0.24	0.07	0.41	4.33	0.10	0.09
63	Rays and skates I	3.27	0.30	0.24	0.07	0.60	3.00	0.76	0.20
64	Benthopelagic cephalopods I	3.53	0.30	0.17	0.05	2.70	9.00	0.93	0.30
65	Benthic cephalopods I	3.42	0.30	0.33	0.10	2.10	7.00	0.95	0.30
66	Bivalves_gastropods I	2.01	0.30	0.70	0.21	1.30	5.00	0.95	0.26
67	Crustaceans I	2.63	0.30	0.63	0.19	3.45	12.00	0.97	0.29
68	Jellyfish I	3.10	0.30	0.17	0.05	11.10	35.90	0.87	0.31
69	Benthos I	2.01	0.30	11.74	3.52	2.75	22.00	0.29	0.13
70	Zooplankton I	2.14	0.30	3.63	1.09	38.44	128.12	0.57	0.30
71	Phytoplankton I	1.00	0.30	7.60	2.28	61.80	--	0.88	--
72	Seagrass I	1.00	0.30	16.00	4.79	2.59	--	0.64	--
73	Piscivores cetaceans E	4.12	0.31	0.00	0.00	0.08	25.84	0.76	0.00
74	Pinnipeds E	4.11	0.31	0.00	0.00	0.08	13.15	0.31	0.01
75	Seabirds E	3.12	0.31	0.00	0.00	4.78	111.61	0.00	0.04
76	Medium Pelagics E	3.23	0.31	0.61	0.19	0.80	4.79	0.92	0.17
77	European pilchard E	3.02	0.31	0.48	0.15	0.95	9.49	0.99	0.10

78	European anchovy E	3.14	0.31	0.87	0.27	0.90	5.20	0.92	0.17
79	Other small pelagics E	2.89	0.31	0.48	0.15	0.95	8.23	0.91	0.12
80	Large demersals E	3.57	0.31	0.18	0.06	0.70	4.35	0.94	0.16
81	European hake E	3.79	0.31	0.28	0.09	0.60	5.26	0.92	0.11
82	Medium demersals E	2.87	0.31	0.40	0.12	1.00	9.09	0.91	0.11
83	Small demersals E	2.95	0.31	0.36	0.11	1.10	7.64	0.99	0.14
84	Deep sea fish E	2.90	0.31	0.42	0.13	0.70	3.50	0.94	0.20
85	Skarks E	3.70	0.31	0.20	0.06	0.50	5.16	0.10	0.10
86	Rays and skates E	3.38	0.31	0.18	0.06	0.70	4.07	0.93	0.17
87	Benthopelagic cephalopods E	3.55	0.31	0.13	0.04	2.70	9.00	0.92	0.30
88	Benthic cephalopods E	3.36	0.31	0.32	0.10	2.10	7.00	0.96	0.30
89	Bivalves_gastropods E	2.01	0.31	0.62	0.19	1.30	5.00	0.98	0.26
90	Crustaceans E	2.64	0.31	0.56	0.17	3.50	12.00	0.98	0.29
91	Jellyfish E	3.25	0.31	0.16	0.05	4.84	15.00	0.75	0.32
92	Benthos E	2.02	0.31	9.83	3.10	2.64	16.13	0.32	0.16
93	Zooplankton E	2.14	0.31	3.59	1.13	38.80	129.33	0.55	0.30
94	Phytoplankton E	1.00	0.31	8.83	2.78	70.00	--	0.66	--
95	Seagrass E	1.00	0.31	15.00	4.72	2.69	--	0.40	--
96	Discards W	1.00	0.33	0.02	0.01	--	--	0.02	--
97	Detritus W	1.00	0.33	32.01	10.63	--	--	0.04	--
98	Discards A	1.00	0.05	0.01	0.00	--	--	0.11	--
99	Detritus A	1.00	0.05	19.73	1.05	--	--	0.25	--
100	Discards I	1.00	0.30	0.01	0.00	--	--	0.27	--
101	Detritus I	1.00	0.30	14.78	4.43	--	--	0.59	--
102	Discards E	1.00	0.31	0.01	0.00	--	--	0.13	--
103	Detritus E	1.00	0.31	14.74	4.64	--	--	0.29	--

S3. Diet composition matrix for the Mediterranean marine ecosystem model for the 1950s period. Prey groups are indicated by rows and predators by columns. Functional group codes are reported according to Table S2.

	1	2	3	4	5	6	7	8	9	10	11	12	13	14	15	16	17	18	19	20	21	22	23	24	27	28	29	30	31	32	33	34	35	36	37	38	39	40	41	42	43	44	45	46	47	50	51	52	53	54	55	56	57	58	59	60	61	62	63	64	65	66	67	68	69	70	73	74	75	76	77	78	79	80	81	82	83	84	85	86	87	88	89	90	91	92	93
1	0	0	0	0	0	0	0	0	0	0	0	0	0	0	0	0	0	0	0	0	0	0	0	0	0	0	0	0	0	0	0	0	0	0	0	0	0	0	0	0	0	0	0	0	0	0	0	0	0	0	0	0	0	0	0	0	0	0	0	0	0	0	0	0	0	0	0	0	0	0	0	0	0	0	0	0	0	0	0	0	0	0	0	0	0	0	0
2	0	0	0	0	0	0	0	0	0	0	0	0	0	0	0	0	0	0	0	0	0	0	0	0	0	0	0	0	0	0	0	0	0	0	0	0	0	0	0	0	0	0	0	0	0	0	0	0	0	0	0	0	0	0	0	0	0	0	0	0	0	0	0	0	0	0	0	0	0	0	0	0	0	0	0	0	0	0	0	0	0	0	0	0	0	0	0
3	0	0	0	0	0	0	0	0	0	0	0	0	0	0	0	0.00	0	0	0	0	0	0	0	0	0	0	0	0	0	0	0	0	0	0	0	0	0	0	0	0	0	0	0	0	0	0	0	0	0	0	0	0	0	0	0	0	0	0	0	0	0	0	0	0	0	0	0	0	0	0	0	0	0	0	0	0	0	0	0	0	0	0	0	0	0	0	0
4	0	0	0	0	0	0	0	0	0	0	0	0	0	0	0	0	0	0	0	0	0	0	0	0	0	0	0	0	0	0	0	0	0	0	0	0	0	0	0	0	0	0	0	0	0	0	0	0	0	0	0	0	0	0	0	0	0	0	0	0	0	0	0	0	0	0	0	0	0	0	0	0	0	0	0	0	0	0	0	0	0	0	0	0	0	0	0
5	0	0	0.00	0	0	0	0	0	0	0	0	0	0	0	0	0.00	0	0	0	0	0	0	0	0	0	0	0	0	0	0	0	0	0	0	0	0	0.00	0	0	0	0	0	0	0	0	0	0	0	0	0	0	0	0	0	0	0	0	0.00	0	0	0	0	0	0	0	0	0	0	0	0	0	0	0	0	0	0	0	0.00	0	0	0	0	0	0	0	0
6	0	0	0	0	0	0	0	0	0	0	0	0	0	0	0	0	0	0	0	0	0	0	0	0	0	0	0	0	0	0	0	0	0	0	0	0	0	0	0	0	0	0	0	0	0	0	0	0	0	0	0	0	0	0	0	0	0	0	0	0	0	0	0	0	0	0	0	0	0	0	0	0	0	0	0	0	0	0	0	0	0	0	0	0	0	0	0
7	0	0	0	0	0	0	0	0	0	0	0	0	0	0	0	0	0	0	0	0	0	0	0	0	0	0	0	0	0	0	0	0	0	0	0	0	0	0	0	0	0	0	0	0	0	0	0	0	0	0	0	0	0	0	0	0	0	0	0	0	0	0	0	0	0	0	0	0	0	0	0	0	0	0	0	0	0	0	0	0	0	0	0	0	0	0	0
8	0.1	0	0	0.2	0	0.1	0	0	0	0	0	0.2	0	0	0	0	0	0	0	0	0	0	0	0	0	0	0	0	0	0	0	0	0	0	0	0	0	0	0	0	0	0	0	0	0	0	0	0	0	0	0	0	0	0	0	0	0	0	0	0	0	0	0	0	0	0	0	0	0	0	0	0	0	0	0	0	0	0	0	0	0	0	0	0	0	0	0
9	0.1	0	0	0.1	0	0.1	0.1	0	0	0	0	0.1	0	0	0	0	0	0	0	0	0	0	0	0	0	0	0	0	0	0	0	0	0	0	0	0	0	0	0	0	0	0	0	0	0	0	0	0	0	0	0	0	0	0	0	0	0	0	0	0	0	0	0	0	0	0	0	0	0	0	0	0	0	0	0	0	0	0	0	0	0	0	0	0	0	0	0
10	0	0	0	0	0	0	0	0	0	0	0.1	0.1	0	0	0	0	0	0	0	0	0	0	0	0	0	0	0	0	0	0	0	0	0	0	0	0	0	0	0	0	0	0	0	0	0	0	0	0	0	0	0	0	0	0	0	0	0	0	0	0	0	0	0	0	0	0	0	0	0	0	0	0	0	0	0	0	0	0	0	0	0	0	0	0	0	0	0
11	0	0	0.1	0	0	0	0	0	0	0	0	0.1	0	0	0	0	0.1	0	0	0	0	0	0	0	0	0	0	0	0	0	0	0	0	0	0	0	0	0	0	0	0	0	0	0	0	0	0	0	0	0	0	0	0	0	0	0	0	0	0	0	0	0	0	0	0	0	0	0	0	0	0	0	0	0	0	0	0	0	0	0	0	0	0	0	0	0	0
12	0.1	0	0	0	0	0	0	0	0	0	0.1	0.1	0	0	0	0	0	0	0	0	0	0	0	0	0	0	0	0	0	0	0	0	0	0	0	0	0	0	0	0	0	0	0	0	0	0	0	0	0	0	0	0	0	0	0	0	0	0	0	0	0	0	0	0	0	0	0	0	0	0	0	0	0	0	0	0	0	0	0	0	0	0	0	0	0	0	0
13	0	0	0.2	0	0	0	0	0	0	0	0.1	0	0	0	0	0	0	0.1	0	0	0	0	0	0	0	0	0	0	0	0	0	0	0	0	0	0	0	0	0	0	0	0	0	0	0	0	0	0	0	0	0	0	0	0	0	0	0	0	0	0	0	0	0	0	0	0	0	0	0	0	0	0	0	0	0	0	0	0	0	0	0	0	0	0	0	0	0
14	0	0	0	0	0	0	0	0	0	0	0.1	0	0	0	0	0.1	0	0.1	0	0	0	0	0	0	0	0	0	0	0	0	0	0	0	0	0	0	0	0	0	0	0	0	0	0	0	0	0	0	0	0	0	0	0	0	0	0	0	0	0	0	0	0	0	0	0	0	0	0	0	0	0	0	0	0	0	0	0	0	0	0	0	0	0	0	0	0	0
15	0.2	0	0	0	0	0	0	0	0	0	0	0	0	0	0	0	0	0.1	0	0	0	0	0	0	0	0	0	0	0	0	0	0	0	0	0	0	0	0	0	0	0	0	0	0	0	0	0	0	0	0	0	0	0	0	0	0	0	0	0	0	0	0	0	0	0	0	0	0	0	0	0	0	0	0	0	0	0	0	0	0	0	0	0	0	0	0	0
16	0	0	0	0	0	0	0	0	0	0	0	0	0	0	0	0	0	0	0	0	0	0	0	0	0	0	0	0	0	0	0	0	0	0	0	0	0	0	0	0	0	0	0	0	0	0	0	0	0	0	0	0	0	0	0	0	0	0	0	0	0	0	0	0	0	0	0	0	0	0	0	0	0	0	0	0	0	0	0	0	0	0	0	0	0	0	0
17	0	0	0	0	0	0	0	0	0	0	0	0	0	0	0	0.1	0	0	0	0	0	0	0	0	0	0	0	0	0	0	0	0	0	0	0	0	0	0	0	0	0	0	0	0	0	0	0	0	0	0	0	0	0	0	0	0	0	0	0	0	0	0	0	0	0	0	0	0	0	0	0	0	0	0	0	0	0	0	0	0	0	0	0	0	0	0	0
18	0.3	0.1	0.1	0	0	0	0	0	0	0	0	0	0	0	0	0.1	0	0	0	0	0	0	0	0	0	0	0	0	0	0	0	0	0	0	0	0	0	0	0	0	0	0	0	0	0	0	0	0	0	0	0	0	0	0	0	0	0	0	0	0	0	0	0	0	0	0	0	0	0	0	0	0	0	0	0	0	0	0	0	0	0	0	0	0	0	0	0
19	0.1	0	0.4	0	0	0	0	0	0	0	0.1	0	0	0	0	0.1	0.1	0.1	0.1	0	0	0	0	0	0	0	0	0	0	0	0	0	0	0	0	0	0	0	0	0	0	0	0	0	0	0	0	0	0	0	0	0	0	0	0	0	0	0	0	0	0	0	0	0	0	0	0	0	0	0	0	0	0	0	0	0	0	0	0	0	0	0	0	0	0	0	0
20	0	0	0.1	0	0	0	0	0	0	0	0	0.1	0	0	0	0.1	0	0	0.2	0	0	0	0	0	0	0	0	0	0	0	0	0	0	0	0	0	0	0	0	0	0	0	0	0	0	0	0	0	0	0	0	0	0	0	0	0	0	0	0	0	0	0	0	0	0	0	0	0	0	0	0	0	0	0	0	0	0	0	0	0	0	0	0	0	0	0	0
21	0.1	0	0.1	0	0	0	0	0	0	0	0.2	0.1	0.1	0.1	0	0.1	0.1	0.4	0.3	0	0	0	0	0	0	0	0	0	0	0	0	0	0	0	0	0	0	0	0	0	0	0	0	0	0	0	0	0	0	0	0	0	0	0	0	0	0	0	0	0	0	0	0	0	0	0	0	0	0	0	0	0	0	0	0	0	0	0	0	0	0	0	0	0	0	0	0
22	0	0	0	0	0	0.1	0	0	0	0	0	0	0	0	0	0	0	0	0	0	0	0.1	0	0	0	0	0	0	0	0	0	0	0	0	0	0	0	0	0	0	0	0	0	0	0	0	0	0	0	0	0	0	0	0	0	0	0	0	0	0	0	0	0	0	0	0	0	0	0	0	0	0	0	0	0	0	0	0	0	0	0	0	0	0	0	0	0
23	0	0	0	0.1	0.1	0	0.4	0	0	0	0.3	0.1	0.5	0.4	0.2	0.2	0.5	0	0.3	0	0.4	0	0	0	0	0	0	0	0	0	0	0	0	0	0	0	0	0	0	0	0	0	0	0	0	0	0	0	0	0	0	0	0	0	0	0	0	0	0	0	0	0	0	0	0	0	0	0	0	0	0	0	0	0	0	0	0	0	0	0	0	0	0	0	0	0	0
24	0	0.3	0	0	0	0	0.5	0.9	1	0.9	0.1	0.1	0.1	0.3	0.6	0	0	0.3	0.1	0	0.2	0.7	0	0.2	0	0	0	0	0	0	0	0	0	0	0	0	0	0	0	0	0	0	0	0	0	0	0	0	0	0	0	0	0	0	0	0	0	0	0	0	0	0	0	0	0	0	0	0	0	0	0	0	0	0	0	0	0	0	0	0	0	0	0	0	0	0	0
25	0	0	0	0	0	0	0	0.1	0	0.1	0	0	0	0	0.1	0	0	0	0	0	0	0.1	0	0.8	0	0	0	0	0	0	0	0	0	0	0	0	0	0	0	0	0	0	0	0	0	0	0	0	0	0	0	0	0	0	0	0	0	0	0	0	0	0	0	0	0	0	0	0	0	0	0	0	0	0	0	0	0	0	0	0	0	0	0	0	0	0	0
26	0	0	0	0	0.1	0	0	0	0	0	0	0	0	0	0	0	0	0	0	0.2	0	0	0.1	0	0	0	0	0	0	0	0	0	0	0	0	0	0	0	0	0	0	0	0	0	0	0	0	0	0	0	0	0	0	0	0	0	0	0	0	0	0	0	0	0	0	0	0	0	0	0	0	0	0	0	0	0	0	0	0	0	0	0	0	0	0	0	0
27	0	0	0	0	0	0	0	0	0	0	0	0	0	0	0	0	0	0	0	0	0	0	0	0	0	0	0	0	0	0	0	0	0	0	0	0	0.00	0	0	0	0	0	0	0	0	0	0	0	0	0	0	0	0	0	0	0	0	0	0	0	0	0	0	0	0	0	0	0	0	0	0	0	0	0	0	0	0	0	0	0	0	0	0	0	0	0	0
28	0	0	0	0	0	0	0	0	0	0	0	0	0	0	0	0	0	0	0	0	0	0	0	0	0	0	0	0	0	0	0	0	0	0	0	0	0.00	0	0	0	0	0	0	0	0	0	0	0	0	0	0	0	0	0	0	0	0	0	0	0	0	0	0	0	0	0	0	0	0	0	0	0	0	0	0	0	0	0	0	0	0	0	0	0	0	0	0
29	0	0	0	0	0	0	0	0	0	0	0	0	0	0	0	0	0	0	0	0	0	0	0	0	0	0	0	0	0	0	0	0	0	0	0	0	0	0	0	0	0	0	0	0	0	0	0	0	0	0	0	0	0	0	0	0	0	0	0	0	0	0	0	0	0	0	0	0	0	0	0	0	0	0	0	0	0	0	0	0	0	0	0	0	0	0	0
30	0	0	0	0	0	0	0	0	0	0	0	0	0	0	0	0	0	0	0	0	0	0	0	0	0.1	0	0	0	0	0	0	0.1	0	0	0	0	0	0	0	0	0	0	0	0	0	0	0	0	0	0	0	0	0	0	0	0	0	0	0	0	0	0	0	0	0	0	0	0	0	0	0	0	0	0	0	0	0	0	0	0	0	0	0	0	0	0	0
31	0	0	0	0	0	0	0	0	0	0	0	0	0	0	0	0	0	0	0	0	0	0	0	0	0.1	0	0.2	0.1	0	0	0	0	0.2	0	0	0	0	0	0	0	0	0	0	0	0	0	0	0	0	0	0	0	0	0	0	0	0	0	0	0	0	0	0	0	0	0	0	0	0	0	0	0	0	0	0	0	0	0	0	0	0	0	0	0	0	0	0
32	0	0	0	0	0	0	0	0	0	0	0	0	0	0	0	0	0	0	0	0	0	0	0	0	0.1	0	0.1	0.1	0	0	0	0	0.1	0	0	0	0	0	0	0	0	0	0	0	0	0	0	0	0	0	0	0	0	0	0	0	0	0	0	0	0	0	0	0	0	0	0	0	0	0	0	0	0	0	0	0	0	0	0	0	0	0	0	0	0	0	0
33	0	0	0	0	0	0	0	0	0	0	0	0	0	0	0	0	0	0	0	0	0	0	0	0	0.1	0	0	0	0	0	0	0	0	0	0	0	0	0	0	0	0	0	0.00	0	0	0	0	0	0	0	0	0	0	0	0	0	0	0	0	0	0	0	0	0	0	0	0	0	0	0	0	0	0	0	0	0	0	0	0	0	0	0	0	0	0	0	0
34	0	0	0	0	0	0	0	0	0	0	0	0	0	0	0	0	0	0	0	0	0	0	0	0	0	0.1	0	0	0	0	0	0	0.1	0	0	0	0	0.1	0	0	0	0	0	0	0	0	0	0	0	0	0	0	0	0	0	0	0	0	0	0	0	0	0	0	0	0	0	0	0	0	0	0	0	0	0	0	0	0	0	0	0	0	0	0	0	0	0
35	0	0	0	0	0	0	0	0	0	0	0	0	0	0	0	0	0	0	0	0	0	0	0	0	0.1	0	0	0	0	0	0	0	0.1	0	0	0	0	0	0	0	0	0	0	0	0	0	0	0	0	0	0	0	0	0	0	0	0	0	0	0	0	0	0	0	0	0	0	0	0	0	0	0	0	0	0	0	0	0	0	0	0	0	0	0	0	0	0
36	0	0	0	0	0	0	0	0	0	0	0	0	0	0	0	0	0	0	0	0	0	0	0	0	0	0.2	0	0	0	0	0	0.1	0	0	0	0	0	0	0.1	0	0	0	0.00	0	0	0	0	0	0	0	0	0	0	0	0	0	0	0	0	0	0	0	0	0	0	0	0	0	0	0	0	0	0	0	0	0	0	0	0	0	0	0	0	0	0	0	0
37	0	0	0	0	0	0	0	0	0	0	0	0	0	0	0	0	0	0	0	0	0	0	0	0	0	0	0	0	0	0	0	0.1	0	0	0	0	0.1	0	0.1	0	0	0	0	0	0	0	0	0	0	0	0	0	0	0	0	0	0	0	0	0	0	0	0	0	0	0	0	0	0	0	0	0	0	0	0	0	0	0	0	0	0	0	0	0	0	0	0
38	0	0	0	0	0	0	0	0	0	0	0	0	0	0	0	0	0	0	0	0	0	0	0	0	0	0	0	0	0	0	0	0	0	0	0	0	0	0	0.1	0	0	0	0	0	0	0	0	0	0	0	0	0	0	0	0	0	0	0	0	0	0	0	0	0	0	0	0	0	0	0	0	0	0	0	0	0	0	0	0	0	0	0	0	0	0	0	0
39	0	0	0	0	0	0	0	0	0	0	0	0	0	0	0	0	0	0	0	0	0	0	0	0	0	0	0	0	0	0	0	0	0	0	0	0	0	0	0	0	0	0	0	0	0	0	0	0	0	0	0	0	0	0	0	0	0	0	0	0	0	0	0	0	0	0	0	0	0	0	0	0	0	0	0	0	0	0	0	0	0	0	0	0	0	0	0
40	0	0	0	0	0	0	0	0	0	0	0	0	0	0	0	0	0	0	0	0	0	0	0	0	0	0	0	0	0	0	0	0	0	0	0	0	0.1	0	0	0	0	0	0	0	0	0	0	0	0	0	0	0	0	0	0	0	0	0	0	0	0	0	0	0	0	0	0	0	0	0	0	0	0	0	0	0	0	0	0	0	0	0	0	0	0	0	0
41	0	0	0	0	0	0	0	0	0	0	0	0	0	0	0	0	0	0	0	0	0	0	0	0	0.3	0.1	0	0	0	0	0	0	0	0	0	0	0.1	0.1	0	0.1	0	0	0	0	0	0	0	0	0	0	0	0	0	0	0	0	0	0	0	0	0	0	0	0	0	0	0	0	0	0	0	0	0	0	0	0	0	0	0	0	0	0	0	0	0	0	0
42	0	0	0	0	0	0	0	0	0	0	0	0	0	0	0	0	0	0	0	0	0	0	0	0	0.1	0.4	0	0	0	0	0	0.1	0.1	0	0	0	0.2	0.1	0	0	0	0	0	0	0	0	0	0	0	0	0	0	0	0	0	0	0	0	0	0	0	0	0	0	0	0	0	0	0	0	0	0	0	0	0	0	0	0	0	0	0	0	0	0	0	0	0
43	0	0	0	0	0	0	0	0	0	0	0	0	0	0	0	0	0	0	0	0	0	0	0	0	0	0.1	0	0	0	0	0	0	0.1	0	0	0	0.1	0	0.1	0.3	0.1	0	0.00	0	0	0	0	0	0	0	0	0	0	0	0	0	0	0	0	0	0	0	0	0	0	0	0	0	0	0	0	0	0	0	0	0	0	0	0	0	0	0	0	0	0	0	0
44	0	0	0	0	0	0	0	0	0	0	0	0	0	0	0	0	0	0	0	0	0	0	0	0	0.1	0.1	0	0	0	0	0	0.2	0.1	0.1	0	0	0.1	0.1	0.4	0.3	0	0	0	0	0	0	0	0	0	0	0	0	0	0	0	0	0	0	0	0	0	0	0	0	0	0	0	0	0	0	0	0	0	0	0	0	0	0	0	0	0	0	0	0	0	0	0
45	0	0	0	0	0	0	0	0	0	0	0	0	0	0	0	0	0	0	0	0	0	0	0	0	0	0	0	0	0	0	0	0	0	0	0	0	0	0	0	0	0	0	0.3	0	0	0	0	0	0	0	0	0	0	0	0	0	0	0	0	0	0	0	0	0	0	0	0	0	0	0	0	0	0	0	0	0	0	0	0	0	0	0	0	0	0	0	0
46	0	0	0	0	0.1	0	0	0	0	0	0	0	0	0	0	0	0	0	0	0	0	0	0	0	0	0	0.1	0.4	0	0	0	0.3	0.1	0.6	0.6	0.3	0.3	0.5	0	0.2	0	0.5	0	0	0	0	0	0	0	0	0	0	0	0	0	0	0	0	0	0	0	0	0	0	0	0	0	0	0	0	0	0	0	0	0	0	0	0	0	0	0	0	0	0	0	0	0
47	0	0	0	0	0	0.1	0	0	0	0	0	0	0	0	0	0	0	0	0	0	0	0	0	0	0	0	0	0.4	0.9	1	0.9	0.1	0.1	0.1	0.2	0.5	0	0	0.3	0.1	0	0.2	0.5	0	0.1	0	0	0	0	0	0	0	0	0	0	0	0	0	0	0	0	0	0	0	0	0	0	0	0	0	0	0	0	0	0	0	0	0	0	0	0	0	0	0	0	0	0
48	0	0	0	0	0	0	0	0	0	0	0	0	0	0	0	0	0	0	0	0	0	0	0	0	0	0	0	0	0.1	0	0.1	0	0	0	0	0.1	0	0	0	0	0	0	0.2	0	0.9	0	0	0	0	0	0	0	0	0	0	0	0	0	0	0	0	0	0	0	0	0	0	0	0	0	0	0	0	0	0	0	0	0	0	0	0	0	0	0	0	0	0
49	0	0	0	0	0.1	0	0	0	0	0	0	0	0	0	0	0	0	0	0	0	0	0	0	0	0	0	0	0	0	0	0	0	0	0	0	0	0	0	0	0	0.2	0	0	0	0	0	0	0	0	0	0	0	0	0	0	0	0	0	0	0	0	0	0	0	0	0	0	0	0	0	0	0	0	0	0	0	0	0	0	0	0	0	0	0	0	0	0
50	0	0	0	0	0	0	0	0	0	0	0	0	0	0	0	0	0	0	0	0	0	0	0	0	0	0	0	0	0	0	0	0	0	0	0	0	0	0	0	0	0	0	0	0	0	0	0	0	0	0	0	0	0	0	0	0	0	0.00	0	0	0	0	0	0	0	0	0	0	0	0	0	0	0	0	0	0	0	0	0	0	0	0	0	0	0	0	0
51	0	0	0	0	0	0	0	0	0	0	0	0	0	0	0	0	0	0	0	0	0	0	0	0	0	0	0	0	0	0	0	0	0	0	0	0	0	0	0	0	0	0	0	0	0	0	0	0	0	0	0	0	0	0	0	0	0	0.00	0	0	0	0	0	0	0	0	0	0	0	0	0	0	0	0	0	0	0	0	0	0	0	0	0	0	0	0	0
52	0	0	0	0	0	0	0	0	0	0	0	0	0	0	0	0	0	0	0	0	0	0	0	0	0	0	0	0	0	0	0	0	0	0	0	0	0	0	0	0	0	0	0	0	0	0	0	0	0	0	0	0	0	0	0	0	0	0	0	0	0	0	0	0	0	0	0	0	0	0	0	0	0	0	0	0	0	0	0	0	0	0	0	0	0	0	0
53	0	0	0	0	0	0	0	0	0	0	0	0	0	0	0	0	0	0	0	0	0	0	0	0	0	0	0	0	0	0	0	0	0	0	0	0	0	0	0	0	0	0	0	0	0	0.1	0	0	0	0	0	0	0.1	0.1	0	0	0	0	0	0	0	0	0	0	0	0	0	0	0	0	0	0	0	0	0	0	0	0	0	0	0	0	0	0	0	0	0
54	0	0	0	0	0	0.1	0	0	0	0	0	0	0	0	0	0	0	0	0	0	0	0	0	0	0	0	0	0	0	0	0	0	0	0	0	0	0	0	0	0	0	0	0	0	0	0.1	0	0.2	0	0	0	0	0	0.2	0	0	0	0	0	0	0	0	0	0	0	0	0	0	0	0	0	0	0	0	0	0	0	0	0	0	0	0	0	0	0	0	0
55	0	0	0	0	0	0.1	0	0	0	0	0	0	0	0	0	0	0	0	0	0	0	0	0	0	0	0	0	0	0	0	0	0	0	0	0	0	0	0	0	0	0	0	0	0	0	0.1	0	0.1	0	0	0	0	0	0.1	0	0	0	0	0	0	0	0	0	0	0	0	0	0	0	0	0	0	0	0	0	0	0	0	0	0	0	0	0	0	0	0	0
56	0	0	0	0	0	0	0	0	0	0	0	0	0	0	0	0	0	0	0	0	0	0	0	0	0	0	0	0	0	0	0	0	0	0	0	0	0	0	0	0	0	0	0	0	0	0.1	0	0	0	0	0	0	0.1	0.1	0	0	0	0	0	0	0	0	0	0	0	0	0	0	0	0	0	0	0	0	0	0	0	0	0	0	0	0	0	0	0	0	0
57	0	0	0	0	0	0	0	0	0	0	0	0	0	0	0	0	0	0	0	0	0	0	0	0	0	0	0	0	0	0	0	0	0	0	0	0	0	0	0	0	0	0	0	0	0	0	0.1	0	0	0	0	0	0	0.1	0	0	0	0	0.1	0	0	0	0	0	0	0	0	0	0	0	0	0	0	0	0	0	0	0	0	0	0	0	0	0	0	0	0
58	0	0	0	0	0	0	0	0	0	0	0	0	0	0	0	0	0	0	0	0	0	0	0	0	0	0	0	0	0	0	0	0	0	0	0	0	0	0	0	0	0	0	0	0	0	0.1	0	0	0	0	0	0	0.1	0.1	0	0	0	0	0	0	0	0	0	0	0	0	0	0	0	0	0	0	0	0	0	0	0	0	0	0	0	0	0	0	0	0	0
59	0	0	0	0	0	0	0	0	0	0	0	0	0	0	0	0	0	0	0	0	0	0	0	0	0	0	0	0	0	0	0	0	0	0	0	0	0	0	0	0	0	0	0	0	0	0	0.2	0	0	0	0	0	0.1	0	0	0	0	0.1	0	0.1	0	0	0	0	0	0	0	0	0	0	0	0	0	0	0	0	0	0	0	0	0	0	0	0	0	0	0
60	0	0	0	0	0	0	0	0	0	0	0	0	0	0	0	0	0	0	0	0	0	0	0	0	0	0	0	0	0	0	0	0	0	0	0	0	0	0	0	0	0	0	0	0	0	0	0	0	0	0	0	0	0.1	0	0	0	0	0.1	0	0	0	0	0	0	0	0	0	0	0	0	0	0	0	0	0	0	0	0	0	0	0	0	0	0	0	0	0
61	0	0	0	0	0	0	0	0	0	0	0	0	0	0	0	0	0	0	0	0	0	0	0	0	0	0	0	0	0	0	0	0	0	0	0	0	0	0	0	0	0	0	0	0	0	0	0	0	0	0	0	0	0	0	0	0	0	0	0	0.2	0	0	0	0	0	0	0	0	0	0	0	0	0	0	0	0	0	0	0	0	0	0	0	0	0	0	0
62	0	0	0	0	0	0	0	0	0	0	0	0	0	0	0	0	0	0	0	0	0	0	0	0	0	0	0	0	0	0	0	0	0	0	0	0	0	0	0	0	0	0	0	0	0	0	0	0	0	0	0	0	0	0	0	0	0	0	0	0	0	0	0	0	0	0	0	0	0	0	0	0	0	0	0	0	0	0	0	0	0	0	0	0	0	0	0
63	0	0	0	0	0	0	0	0	0	0	0	0	0	0	0	0	0	0	0	0	0	0	0	0	0	0	0	0	0	0	0	0	0	0	0	0	0	0	0	0	0	0	0	0	0	0	0	0	0	0	0	0	0	0	0	0	0	0.1	0	0	0	0	0	0	0	0	0	0	0	0	0	0	0	0	0	0	0	0	0	0	0	0	0	0	0	0	0
64	0	0.1	0	0	0	0	0	0	0	0	0	0	0	0	0	0	0	0	0	0	0	0	0	0	0	0	0	0	0	0	0	0	0	0	0	0	0	0	0	0	0	0	0	0	0	0.3	0.1	0	0	0	0	0	0	0	0	0	0	0.1	0.1	0	0	0	0	0	0	0	0	0	0	0	0	0	0	0	0	0	0	0	0	0	0	0	0	0	0	0	0
65	0	0	0	0	0	0	0	0	0	0	0	0	0	0	0	0	0	0	0	0	0	0	0	0	0	0	0	0	0	0	0	0	0	0	0	0	0	0	0	0	0	0	0	0	0	0.1	0.5	0	0	0	0	0	0.1	0	0	0	0	0.1	0.1	0	0.1	0	0	0	0	0	0	0	0	0	0	0	0	0	0	0	0	0	0	0	0	0	0	0	0	0	0
66	0	0	0	0	0	0	0	0	0	0	0	0	0	0	0	0	0	0	0	0	0	0	0	0	0	0	0	0	0	0	0	0	0	0	0	0	0	0	0	0	0	0	0	0	0	0	0.1	0	0	0	0	0	0	0.1	0	0	0	0.1	0	0.1	0.2	0	0	0.00	0	0	0	0	0	0	0	0	0	0	0	0	0	0	0	0	0	0	0	0	0	0	0
67	0	0	0	0	0	0	0	0	0	0	0	0	0	0	0	0	0	0	0	0	0	0	0	0	0	0	0	0	0	0	0	0	0	0	0	0	0	0	0	0	0	0	0	0	0	0.1	0.1	0	0	0	0	0	0.2	0.1	0.1	0.1	0	0.1	0.1	0.4	0.3	0	0	0	0	0	0	0	0	0	0	0	0	0	0	0	0	0	0	0	0	0	0	0	0	0	0
68	0	0	0	0	0	0	0	0	0	0	0	0	0	0	0	0	0	0	0	0	0	0	0	0	0	0	0	0	0	0	0	0	0	0	0	0	0	0	0	0	0	0	0	0	0	0	0	0.1	0	0	0	0	0	0	0	0	0	0	0	0	0	0	0	0.3	0	0	0	0	0	0	0	0	0	0	0	0	0	0	0	0	0	0	0	0	0	0	0
69	0	0	0	0	0.1	0	0	0	0	0	0	0	0	0	0	0	0	0	0	0	0	0	0	0	0	0	0	0	0	0	0	0	0	0	0	0	0	0	0	0	0	0	0	0	0	0	0	0	0.5	0	0	0	0.3	0.1	0.4	0.4	0.2	0.2	0.3	0	0.2	0	0.4	0	0	0	0	0	0	0	0	0	0	0	0	0	0	0	0	0	0	0	0	0	0	0	0
70	0	0.2	0	0	0	0.1	0	0	0	0	0	0	0	0	0	0	0	0	0	0	0	0	0	0	0	0	0	0	0	0	0	0	0	0	0	0	0	0	0	0	0	0	0	0	0	0	0	0	0.4	0.9	1	0.9	0.1	0.1	0.2	0.3	0.5	0	0	0.3	0.1	0	0.1	0.5	0	0.1	0	0	0	0	0	0	0	0	0	0	0	0	0	0	0	0	0	0	0	0	0
71	0	0	0	0	0	0	0	0	0	0	0	0	0	0	0	0	0	0	0	0	0	0	0	0	0	0	0	0	0	0	0	0	0	0	0	0	0	0	0	0	0	0	0	0	0	0	0	0	0	0.1	0	0.1	0	0	0	0	0.2	0	0	0	0	0	0	0.2	0	0.9	0	0	0	0	0	0	0	0	0	0	0	0	0	0	0	0	0	0	0	0	0
72	0	0	0	0	0.1	0	0	0	0	0	0	0	0	0	0	0	0	0	0	0	0	0	0	0	0	0	0	0	0	0	0	0	0	0	0	0	0	0	0	0	0	0	0	0	0	0	0	0	0	0	0	0	0	0	0	0	0	0	0	0	0	0.2	0	0	0.1	0	0	0	0	0	0	0	0	0	0	0	0	0	0	0	0	0	0	0	0	0	0
73	0	0	0	0	0	0	0	0	0	0	0	0	0	0	0	0	0	0	0	0	0	0	0	0	0	0	0	0	0	0	0	0	0	0	0	0	0	0	0	0	0	0	0	0	0	0	0	0	0	0	0	0	0	0	0	0	0	0	0	0	0	0	0	0	0	0	0	0	0	0	0	0	0	0	0	0	0	0	0.00	0	0	0	0	0	0	0	0
74	0	0	0	0	0	0	0	0	0	0	0	0	0	0	0	0	0	0	0	0	0	0	0	0	0	0	0	0	0	0	0	0	0	0	0	0	0	0	0	0	0	0	0	0	0	0	0	0	0	0	0	0	0	0	0	0	0	0	0	0	0	0	0	0	0	0	0	0	0	0	0	0	0	0	0	0	0	0	0.00	0	0	0	0	0	0	0	0
75	0	0	0	0	0	0	0	0	0	0	0	0	0	0	0	0	0	0	0	0	0	0	0	0	0	0	0	0	0	0	0	0	0	0	0	0	0	0	0	0	0	0	0	0	0	0	0	0	0	0	0	0	0	0	0	0	0	0	0	0	0	0	0	0	0	0	0	0	0	0	0	0	0	0	0	0	0	0	0	0	0	0	0	0	0	0	0
76	0	0	0	0	0	0	0	0	0	0	0	0	0	0	0	0	0	0	0	0	0	0	0	0	0	0	0	0	0	0	0	0	0	0	0	0	0	0	0	0	0	0	0	0	0	0	0	0	0	0	0	0	0	0	0	0	0	0	0	0	0	0	0	0	0	0	0.1	0	0	0	0	0	0	0.1	0.1	0	0	0	0	0	0	0	0	0	0	0	0
77	0	0	0	0	0	0	0	0	0	0	0	0	0	0	0	0	0	0	0	0	0	0	0	0	0	0	0	0	0	0	0	0	0	0	0	0	0	0	0	0	0	0	0	0	0	0	0	0	0	0	0	0	0	0	0	0	0	0	0	0	0	0	0	0	0	0	0.1	0	0.2	0	0	0	0	0	0.1	0	0	0	0	0	0	0	0	0	0	0	0
78	0	0	0	0	0	0.1	0	0	0	0	0	0	0	0	0	0	0	0	0	0	0	0	0	0	0	0	0	0	0	0	0	0	0	0	0	0	0	0	0	0	0	0	0	0	0	0	0	0	0	0	0	0	0	0	0	0	0	0	0	0	0	0	0	0	0	0	0.1	0	0.1	0.1	0	0	0	0	0.1	0	0	0	0	0	0	0	0	0	0	0	0
79	0	0	0	0	0	0	0	0	0	0	0	0	0	0	0	0	0	0	0	0	0	0	0	0	0	0	0	0	0	0	0	0	0	0	0	0	0	0	0	0	0	0	0	0	0	0	0	0	0	0	0	0	0	0	0	0	0	0	0	0	0	0	0	0	0	0	0.1	0	0	0	0	0	0	0.1	0.1	0	0	0	0	0	0	0	0	0	0	0	0
80	0	0	0	0	0	0	0	0	0	0	0	0	0	0	0	0	0	0	0	0	0	0	0	0	0	0	0	0	0	0	0	0	0	0	0	0	0	0	0	0	0	0	0	0	0	0	0	0	0	0	0	0	0	0	0	0	0	0	0	0	0	0	0	0	0	0	0	0.1	0	0	0	0	0	0	0	0	0	0	0	0.1	0	0	0	0	0	0	0
81	0	0	0	0	0	0	0	0	0	0	0	0	0	0	0	0	0	0	0	0	0	0	0	0	0	0	0	0	0	0	0	0	0	0	0	0	0	0	0	0	0	0	0	0	0	0	0	0	0	0	0	0	0	0	0	0	0	0	0	0	0	0	0	0	0	0	0.1	0	0	0	0	0	0	0.1	0.1	0	0	0	0	0	0	0	0	0	0	0	0
82	0	0	0	0	0	0	0	0	0	0	0	0	0	0	0	0	0	0	0	0	0	0	0	0	0	0	0	0	0	0	0	0	0	0	0	0	0	0	0	0	0	0	0	0	0	0	0	0	0	0	0	0	0	0	0	0	0	0	0	0	0	0	0	0	0	0	0	0.2	0	0	0	0	0	0.1	0	0	0	0	0	0	0.1	0	0	0	0	0	0
83	0	0	0	0	0	0	0	0	0	0	0	0	0	0	0	0	0	0	0	0	0	0	0	0	0	0	0	0	0	0	0	0	0	0	0	0	0	0	0	0	0	0	0	0	0	0	0	0	0	0	0	0	0	0	0	0	0	0	0	0	0	0	0	0	0	0	0	0	0	0	0	0	0	0.1	0	0	0	0	0.1	0	0.1	0	0	0	0	0	0
84	0	0	0	0	0	0	0	0	0	0	0	0	0	0	0	0	0	0	0	0	0	0	0	0	0	0	0	0	0	0	0	0	0	0	0	0	0	0	0	0	0	0	0	0	0	0	0	0	0	0	0	0	0	0	0	0	0	0	0	0	0	0	0	0	0	0	0	0	0	0	0	0	0	0	0	0	0	0	0	0	0.2	0	0	0	0	0	0
85	0	0	0	0	0	0	0	0	0	0	0	0	0	0	0	0	0	0	0	0	0	0	0	0	0	0	0	0	0	0	0	0	0	0	0	0	0	0	0	0	0	0	0	0	0	0	0	0	0	0	0	0	0	0	0	0	0	0	0	0	0	0	0	0	0	0	0	0	0	0	0	0	0	0	0	0	0	0	0	0	0	0	0	0	0	0	0
86	0	0	0	0	0	0	0	0	0	0	0	0	0	0	0	0	0	0	0	0	0	0	0	0	0	0	0	0	0	0	0	0	0	0	0	0	0	0	0	0	0	0	0	0	0	0	0	0	0	0	0	0	0	0	0	0	0	0	0	0	0	0	0	0	0	0	0	0	0	0	0	0	0	0	0	0	0	0	0.1	0	0	0	0	0	0	0	0
87	0	0	0	0	0	0	0	0	0	0	0	0	0	0	0	0	0	0	0	0	0	0	0	0	0	0	0	0	0	0	0	0	0	0	0	0	0	0	0	0	0	0	0	0	0	0	0	0	0	0	0	0	0	0	0	0	0	0	0	0	0	0	0	0	0	0	0.3	0.1	0	0	0	0	0	0	0	0	0	0	0.1	0.1	0	0	0	0	0	0	0
88	0	0	0	0	0	0	0	0	0	0	0	0	0	0	0	0	0	0	0	0	0	0	0	0	0	0	0	0	0	0	0	0	0	0	0	0	0	0	0	0	0	0	0	0	0	0	0	0	0	0	0	0	0	0	0	0	0	0	0	0	0	0	0	0	0	0	0.1	0.4	0	0	0	0	0	0.1	0	0	0	0	0.1	0.1	0.1	0	0	0	0	0	0
89	0	0	0	0	0	0	0	0	0	0	0	0	0	0	0	0	0	0	0	0	0	0	0	0	0	0	0	0	0	0	0	0	0	0	0	0	0	0	0	0	0	0	0	0	0	0	0	0	0	0	0	0	0	0	0	0	0	0	0	0	0	0	0	0	0	0	0	0.1	0	0	0	0	0	0	0.1	0	0	0	0.1	0	0.1	0.2	0	0	0.00	0	0
90	0	0	0	0	0	0	0	0	0	0	0	0	0	0	0	0	0	0	0	0	0	0	0	0	0	0	0	0	0	0	0	0	0	0	0	0	0	0	0	0	0	0	0	0	0	0	0	0	0	0	0	0	0	0	0	0	0	0	0	0	0	0	0	0	0	0	0.1	0.1	0	0	0	0	0	0.1	0.1	0.1	0.1	0	0.1	0.1	0.3	0.3	0	0	0	0	0
91	0	0	0	0	0	0	0	0	0	0	0	0	0	0	0	0	0	0	0	0	0	0	0	0	0	0	0	0	0	0	0	0	0	0	0	0	0	0	0	0	0	0	0	0	0	0	0	0	0	0	0	0	0	0	0	0	0	0	0	0	0	0	0	0	0	0	0	0	0.1	0	0	0	0	0	0	0	0	0	0	0	0	0	0	0	0.2	0	0
92	0	0	0	0	0.1	0	0	0	0	0	0	0	0	0	0	0	0	0	0	0	0	0	0	0	0	0	0	0	0	0	0	0	0	0	0	0	0	0	0	0	0	0	0	0	0	0	0	0	0	0	0	0	0	0	0	0	0	0	0	0	0	0	0	0	0	0	0	0	0	0.4	0	0	0	0.4	0.1	0.4	0.4	0.2	0.3	0.5	0	0.3	0	0.4	0	0	0
93	0	0.2	0	0	0	0	0	0	0	0	0	0	0	0	0	0	0	0	0	0	0	0	0	0	0	0	0	0	0	0	0	0	0	0	0	0	0	0	0	0	0	0	0	0	0	0	0	0	0	0	0	0	0	0	0	0	0	0	0	0	0	0	0	0	0	0	0	0	0	0.5	0.9	1	0.8	0.1	0.1	0.1	0.3	0.6	0	0	0.3	0.1	0	0.2	0.7	0	0.1
94	0	0	0	0	0	0	0	0	0	0	0	0	0	0	0	0	0	0	0	0	0	0	0	0	0	0	0	0	0	0	0	0	0	0	0	0	0	0	0	0	0	0	0	0	0	0	0	0	0	0	0	0	0	0	0	0	0	0	0	0	0	0	0	0	0	0	0	0	0	0	0.1	0	0.2	0	0	0	0	0.1	0	0	0	0	0	0	0.1	0	0.9
95	0	0	0	0	0.1	0	0	0	0	0	0	0	0	0	0	0	0	0	0	0	0	0	0	0	0	0	0	0	0	0	0	0	0	0	0	0	0	0	0	0	0	0	0	0	0	0	0	0	0	0	0	0	0	0	0	0	0	0	0	0	0	0	0	0	0	0	0	0	0	0	0	0	0	0	0	0	0	0	0	0	0	0	0.2	0	0	0.1	0
96	0.1	0	0	0.3	0	0	0	0	0	0	0	0.1	0	0	0	0	0.2	0	0	0	0	0	0.1	0	0	0	0	0	0	0	0	0	0	0	0	0	0	0	0	0	0	0	0	0	0	0	0	0	0	0	0	0	0	0	0	0	0	0	0	0	0	0	0	0	0	0	0	0	0	0	0	0	0	0	0	0	0	0	0	0	0	0	0	0	0	0	0
97	0	0	0	0	0	0	0	0	0	0	0	0	0.2	0.2	0.1	0	0	0	0	0.8	0.3	0.1	0.8	0	0	0	0	0	0	0	0	0	0	0	0	0	0	0	0	0	0	0	0	0	0	0	0	0	0	0	0	0	0	0	0	0	0	0	0	0	0	0	0	0	0	0	0	0	0	0	0	0	0	0	0	0	0	0	0	0	0	0	0	0	0	0	0
98	0	0	0	0	0	0	0	0	0	0	0	0	0	0	0	0	0	0	0	0	0	0	0	0	0.1	0	0.3	0	0	0	0	0	0	0	0	0	0	0.1	0	0	0	0	0	0.1	0	0	0	0	0	0	0	0	0	0	0	0	0	0	0	0	0	0	0	0	0	0	0	0	0	0	0	0	0	0	0	0	0	0	0	0	0	0	0	0	0	0	0
99	0	0	0	0	0	0	0	0	0	0	0	0	0	0	0	0	0	0	0	0	0	0	0	0	0	0	0	0	0	0	0	0	0	0.2	0.2	0.1	0	0	0	0	0.8	0.3	0	0.9	0	0	0	0	0	0	0	0	0	0	0	0	0	0	0	0	0	0	0	0	0	0	0	0	0	0	0	0	0	0	0	0	0	0	0	0	0	0	0	0	0	0	0
100	0	0	0	0	0	0	0	0	0	0	0	0	0	0	0	0	0	0	0	0	0	0	0	0	0	0	0	0	0	0	0	0	0	0	0	0	0	0	0	0	0	0	0	0	0	0.1	0	0.3	0	0	0	0	0	0	0	0	0	0	0.2	0	0	0	0	0	0.1	0	0	0	0	0	0	0	0	0	0	0	0	0	0	0	0	0	0	0	0	0	0
101	0	0	0	0	0	0	0	0	0	0	0	0	0	0	0	0	0	0	0	0	0	0	0	0	0	0	0	0	0	0	0	0	0	0	0	0	0	0	0	0	0	0	0	0	0	0	0	0	0	0	0	0	0	0	0.3	0.2	0.1	0	0	0	0	0.8	0.4	0	0.8	0	0	0	0	0	0	0	0	0	0	0	0	0	0	0	0	0	0	0	0	0	0
102	0	0	0	0	0	0	0	0	0	0	0	0	0	0	0	0	0	0	0	0	0	0	0	0	0	0	0	0	0	0	0	0	0	0	0	0	0	0	0	0	0	0	0	0	0	0	0	0	0	0	0	0	0	0	0	0	0	0	0	0	0	0	0	0	0	0	0.1	0	0.3	0	0	0	0	0	0	0	0	0	0	0.1	0	0	0	0	0	0.1	0
103	0	0	0	0	0	0	0	0	0	0	0	0	0	0	0	0	0	0	0	0	0	0	0	0	0	0	0	0	0	0	0	0	0	0	0	0	0	0	0	0	0	0	0	0	0	0	0	0	0	0	0	0	0	0	0	0	0	0	0	0	0	0	0	0	0	0	0	0	0	0	0	0	0	0	0	0.3	0.2	0.1	0	0	0	0	0.8	0.4	0	0.8	0
Import	0	0.1	0	0.3	0.1	0	0	0	0	0	0	0	0	0	0	0.2	0	0	0	0	0	0	0	0	0	0	0.3	0	0	0	0	0	0	0	0	0	0.1	0	0	0	0	0	0	0	0	0	0	0.3	0	0	0	0	0	0	0	0	0	0.2	0	0	0	0	0	0	0	0	0	0	0.3	0	0	0	0	0	0	0	0	0	0	0.2	0	0	0	0	0	0	0
Sum	1	1	1	1	1	1	1	1	1	1	1	1	1	1	1	1	1	1	1	1	1	1	1	1	1	1	1	1	1	1	1	1	1	1	1	1	1	1	1	1	1	1	1	1	1	1	1	1	1	1	1	1	1	1	1	1	1	1	1	1	1	1	1	1	1	1	1	1	1	1	1	1	1	1	1	1	1	1	1	1	1	1	1	1	1	1	1

S4. PREBAL for each Mediterranean sub-area (Western [W]; Adriatic [A]; Ionian [I]; Eastern and Levantine [E] plotting (a) biomass estimates (t/km^2), (b) production/biomass ratio (per year), and (c) consumption/biomass (per year) on a log scale vs trophic level, from lowest to highest trophic level, of each species/functional group.

a)

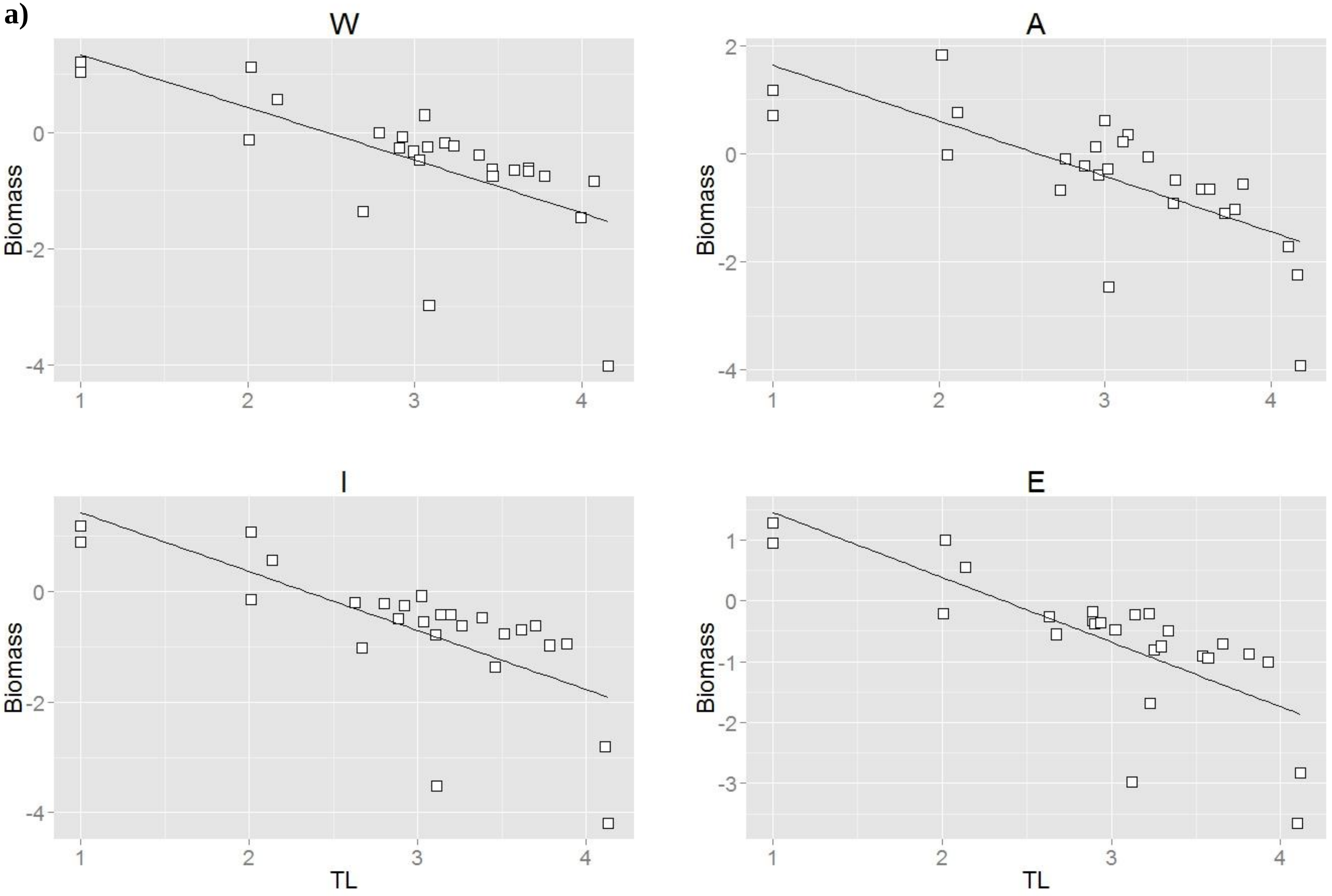

b)

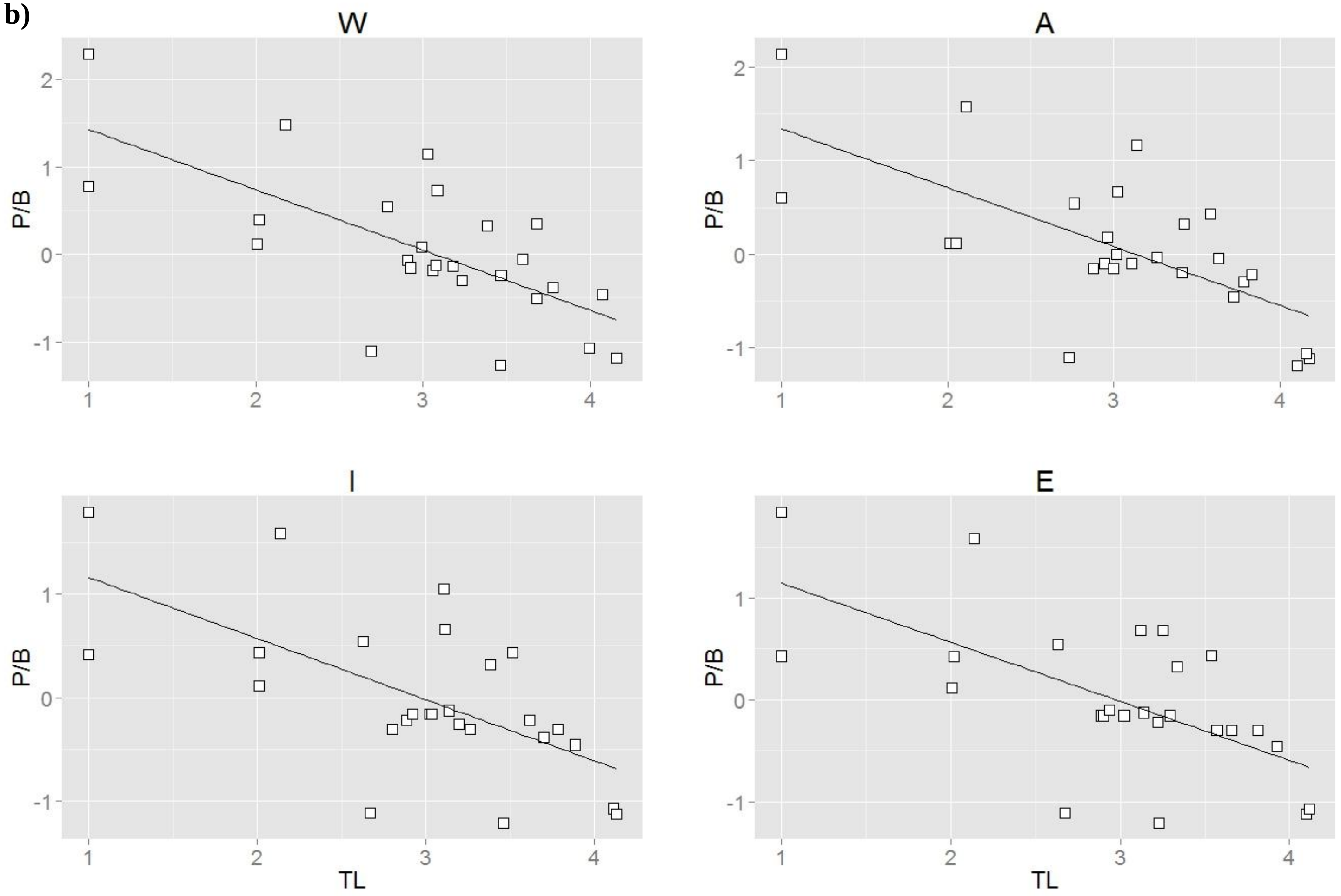

c)

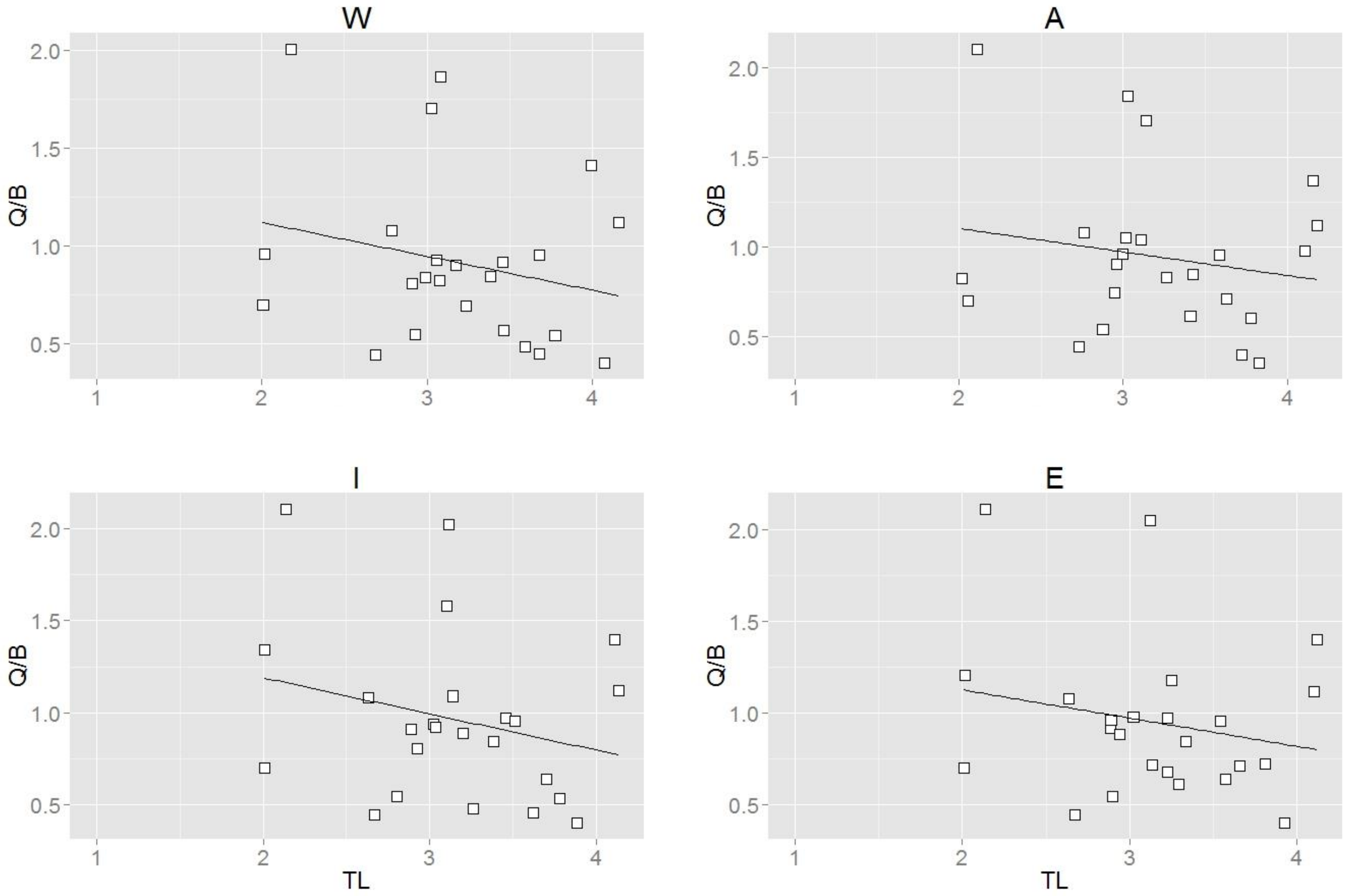

S5. The main drivers used in the fitting procedure: 1) reconstructed fishing effort (kW·days^{-1}) for the main fishing fleets (trawlers: TRWL; purse seiners: PS; long liners: LONG; artisanals: ART) of the four Mediterranean sub-areas (Western: W; Adriatic: A; Ionian: I; Eastern and Levantine: E); 2a) primary production anomaly (PP anom; t·Km^{-2}·year^{-1}) estimated by the model and 2b) primary production from the biogeochemical model (PPbiog; t·Km^{-2}·year^{-1}) for the four Mediterranean sub-areas and the whole Mediterranean Sea.

1)

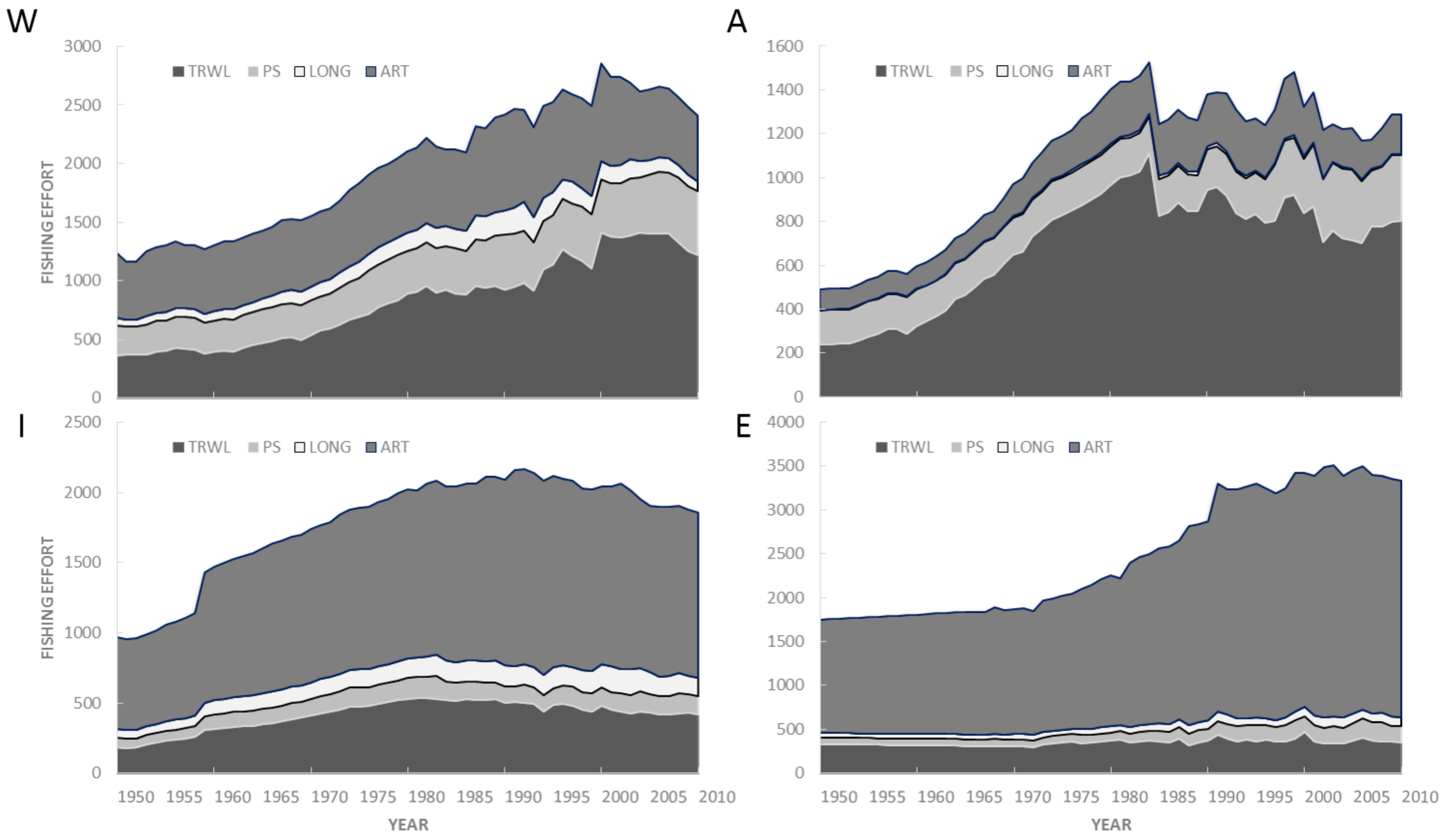

2a) 2b)

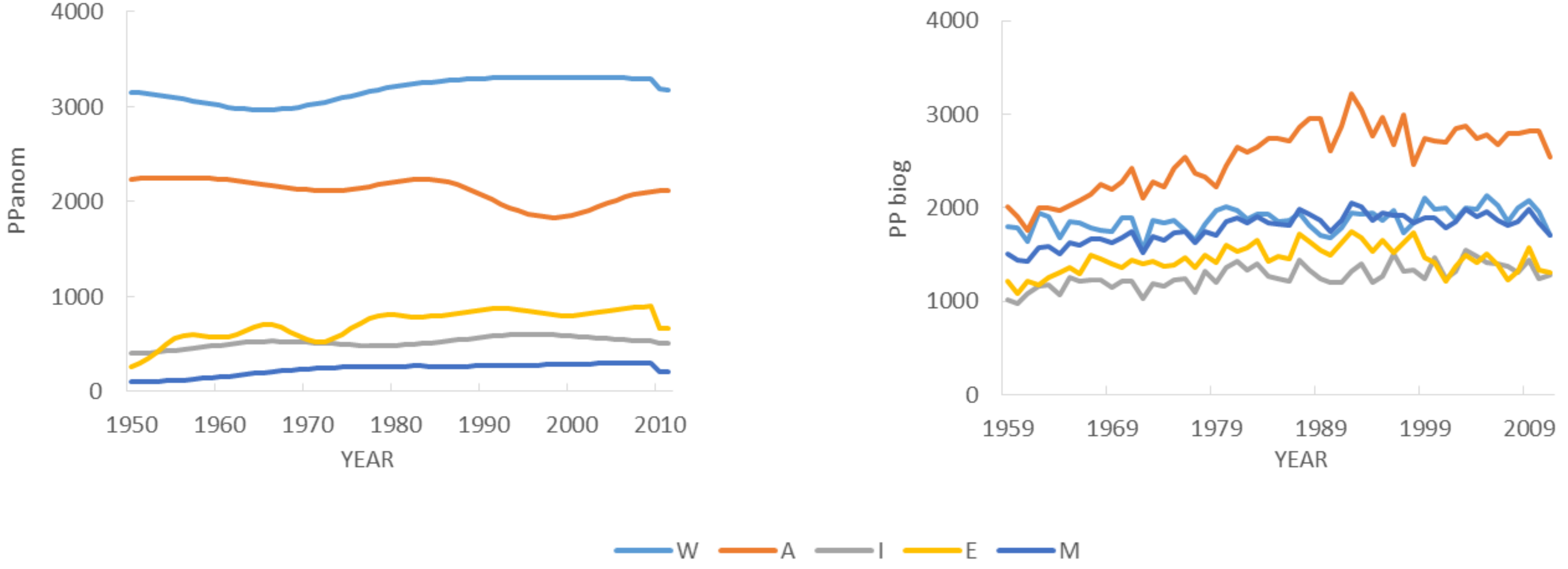

S6. Technological coefficients of fishing vessels by gear type used in the analyses (Sources: [47-49]).

	Technological coefficient		
**Vessel type**	**1950-1979**	**1980-1995**	**1996-2010**
Trawlers	0.5	1	1.8
Purse seiners	0.5	1	1.8
Artisanal	0.5	1	1.3
Longliners	0.5	1	2.8

S7. Graphical presentation of the Spearman correlation analysis. Scatter plots show, for the four sub-areas (Western: W; Adriatic: A; Ionian: I; Eastern and Levantine: E) and for the additional Mediterranean Sea as whole (Mediterranean: M), values of PP from the biogeochemical model (PP biog) versus PP anomaly. Both time series were divided by their mean to be able to compare the two trends.

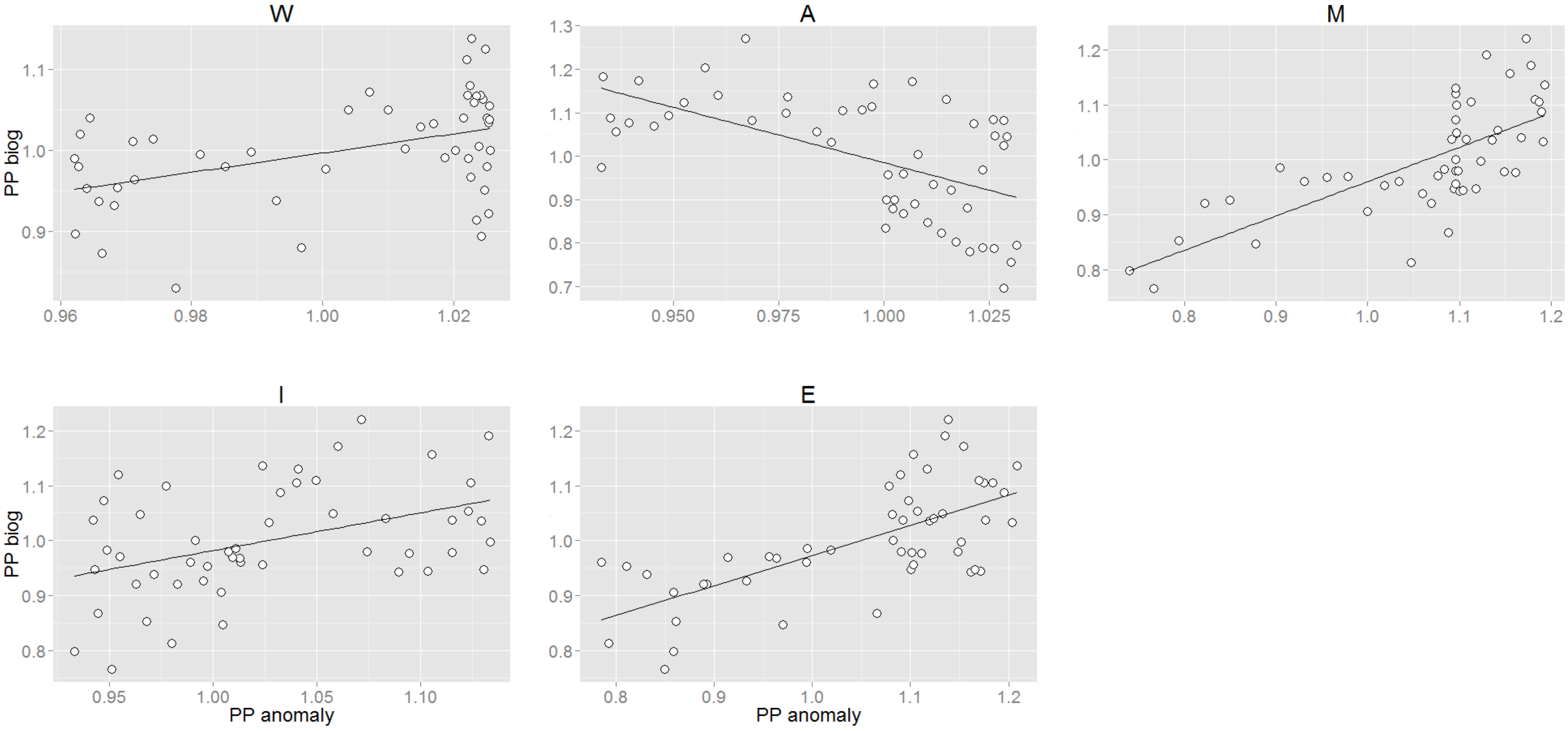

S8. Representation of modelling fitting for the remaining functional groups occurring in the Western (W), Adriatic (A), Ionian (I) and Eastern/Levantine (E) Seas for the period 1950-2011. Predicted biomass (t·km^{-2}) is shown as solid black lines while observed data is represented as black dots. Functional groups codes correspond to those given in Fig 2. The predicted model results (dashed red line) using the modelled biogeochemical PP is also shown. Blue shadow represents the 95% percentile and 5% percentile obtained through the Monte Carlo routine.

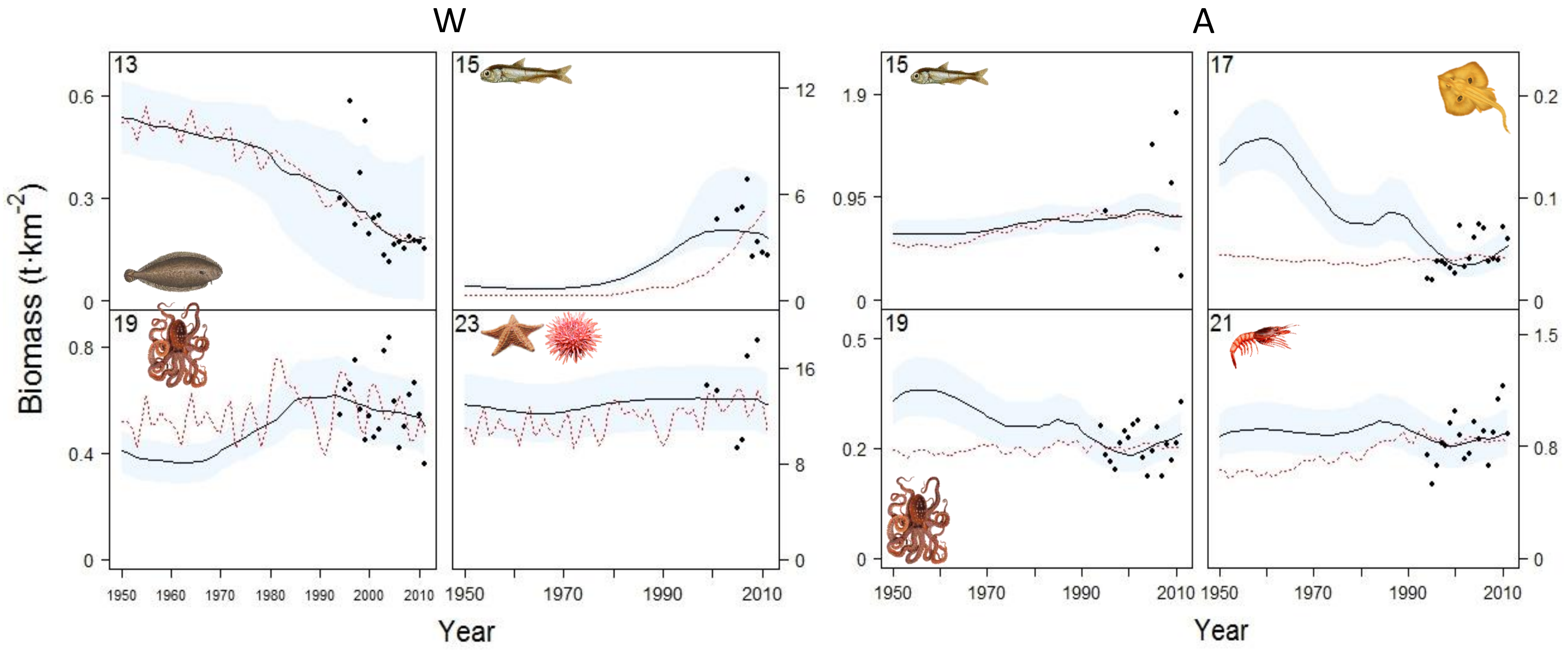

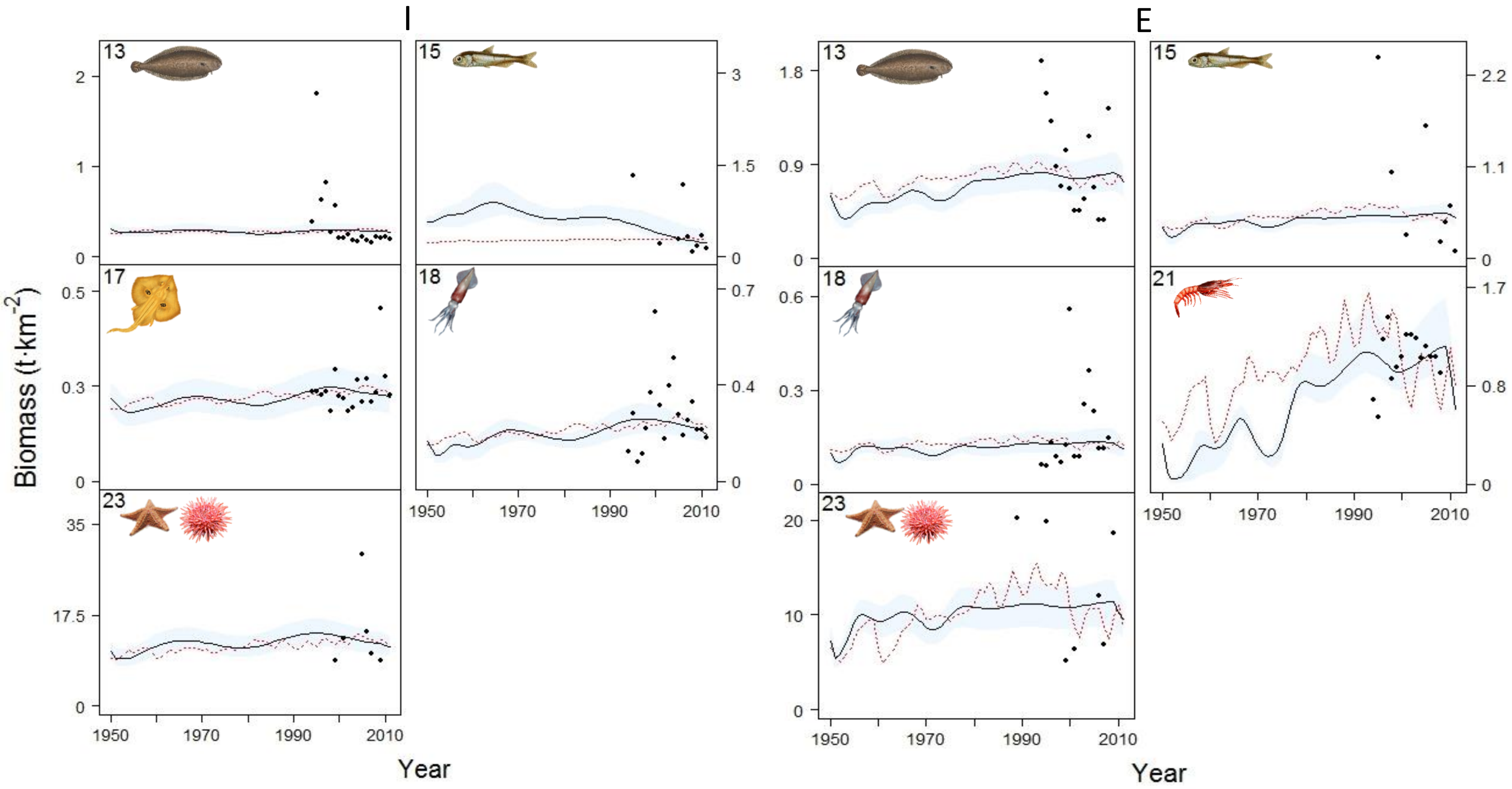
I
E
Biomass (t·km⁻²)
Year
13
15
17
18
21
23
1950
1970
1990
2010

S9. Predicted (solid lines) *versus* observed (dots) catches (t·km^{-2}·year^{-1}) for main commercially important functional groups of the Ionian (c) and Eastern (d) Mediterranean ecosystems (1950-2011). Drawings for species/functional groups 1-3, 5-6 and 8 are by Massimo Demma - http://www.massimodemma.it/-.

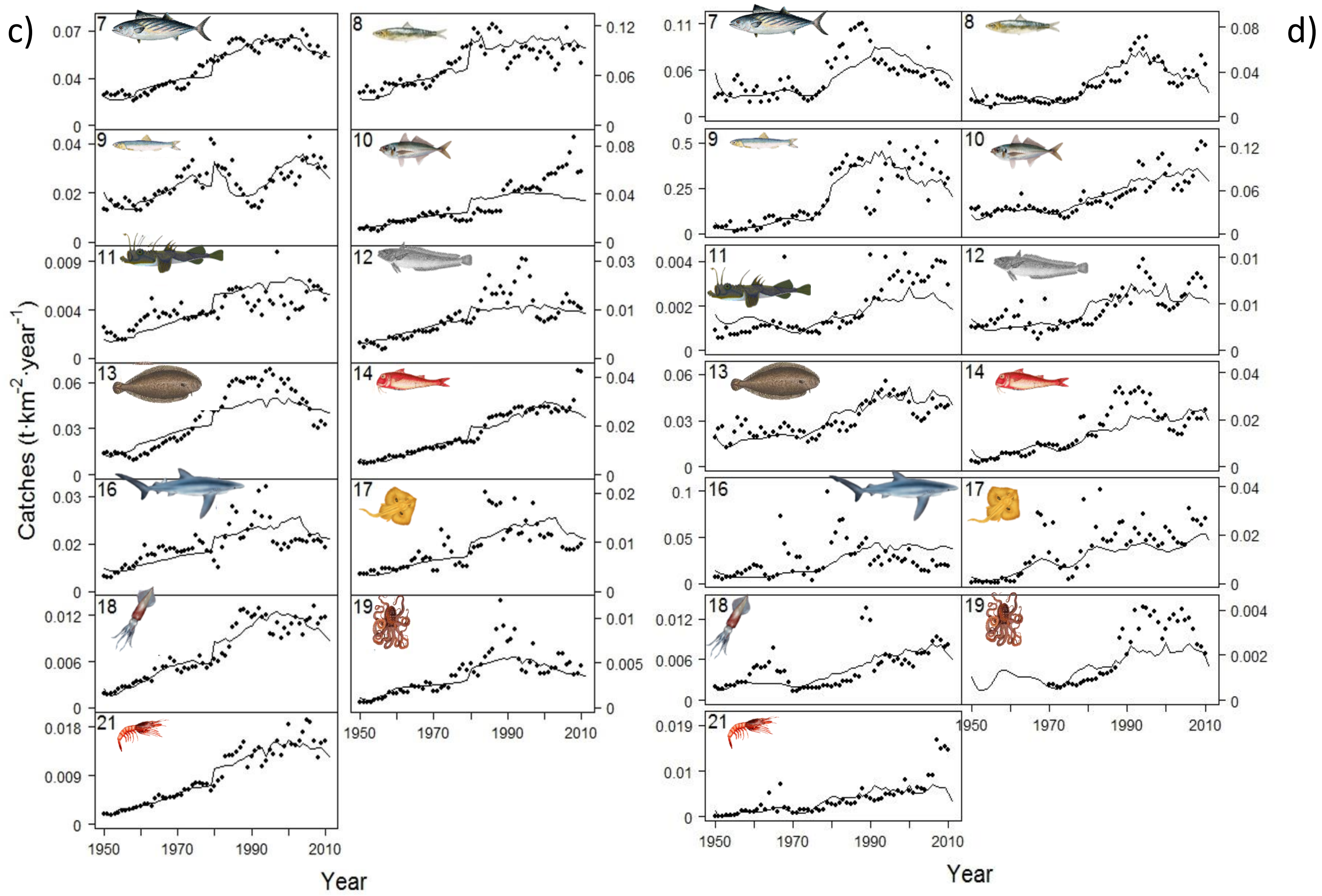

S10. Ecological indicators (1. Forage fish biomass (t·km^{-2}); 2. Demersal fish biomass (t·km^{-2}); 3. Invertebrates biomass (t·km^{-2}); 4. Sharks/rays and skate biomass (t·km^{-2}); 5. Kempton's index of biodiversity; 6. Tot Catch: Total Catch (t· km^{-2} ·year^{-1}); 7. mTLco: Mean trophic level of the community; 8. mTL>3.25: Mean trophic levels of groups having trophic level >3.25 (excluding marine mammals, sea turtles and seabirds); 9 mTLC: Mean trophic level of the catches) estimated from results of the Ecosim model for the period 1950-2011 for the Western Mediterranean Sea. Blue shadow represents the 95% percentile and 5% percentile obtained through the Monte Carlo routine.

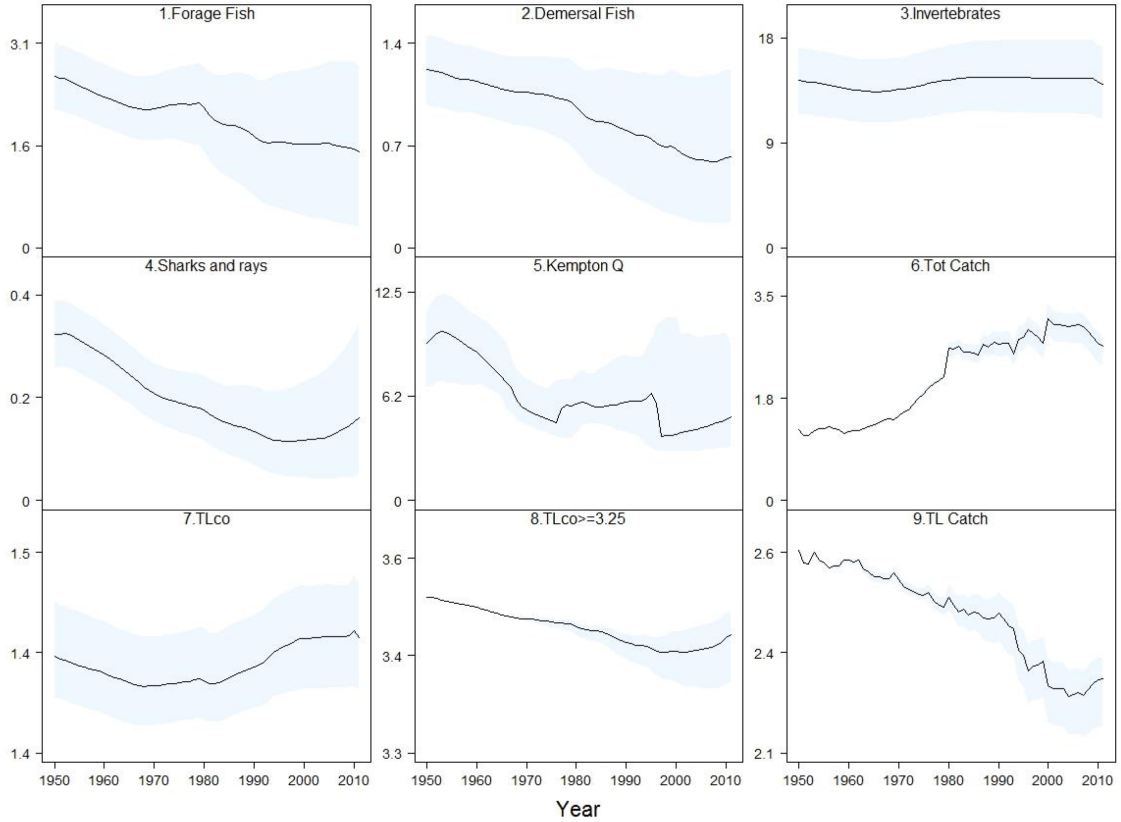

S11. Ecological indicators (1. Forage fish biomass (t·km^{-2}); 2. Demersal fish biomass (t·km^{-2}); 3. Invertebrates biomass (t·km^{-2}); 4. Sharks/rays and skate biomass (t·km^{-2}); 5. Kempton's index of biodiversity; 6. Tot Catch: Total Catch (t· km^{-2} ·year^{-1}); 7. mTLco: Mean trophic level of the community; 8. mTL>3.25: Mean trophic levels of groups having trophic level >3.25 (excluding marine mammals, sea turtles and seabirds); 9 mTLC: Mean trophic level of the catches) estimated from results of the Ecosim model for the period 1950-2011 for the Adriatic Sea. Blue shadow represents the 95% percentile and 5% percentile obtained through the Monte Carlo routine.

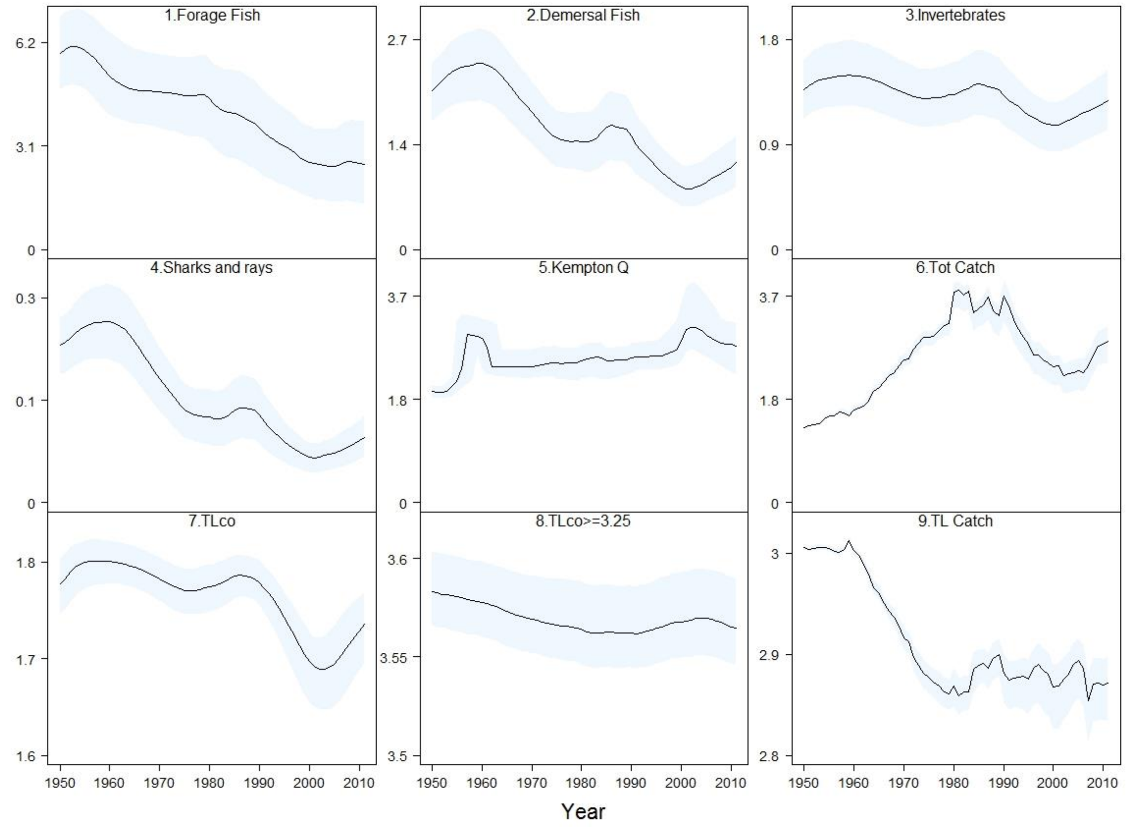

S12. Ecological indicators (1. Forage fish biomass (t·km^{-2}); 2. Demersal fish biomass (t·km^{-2}); 3. Invertebrates biomass (t·km^{-2}); 4. Sharks/rays and skate biomass (t·km^{-2}); 5. Kempton's index of biodiversity; 6. Tot Catch: Total Catch (t· km^{-2} ·year^{-1}); 7. mTLco: Mean trophic level of the community; 8. mTL>3.25: Mean trophic levels of groups having trophic level >3.25 (excluding marine mammals, sea turtles and seabirds); 9 mTLC: Mean trophic level of the catches) estimated from results of the Ecosim model for the period 1950-2011 for the Ionian Sea Sea. Blue shadow represents the 95% percentile and 5% percentile obtained through the Monte Carlo routine.

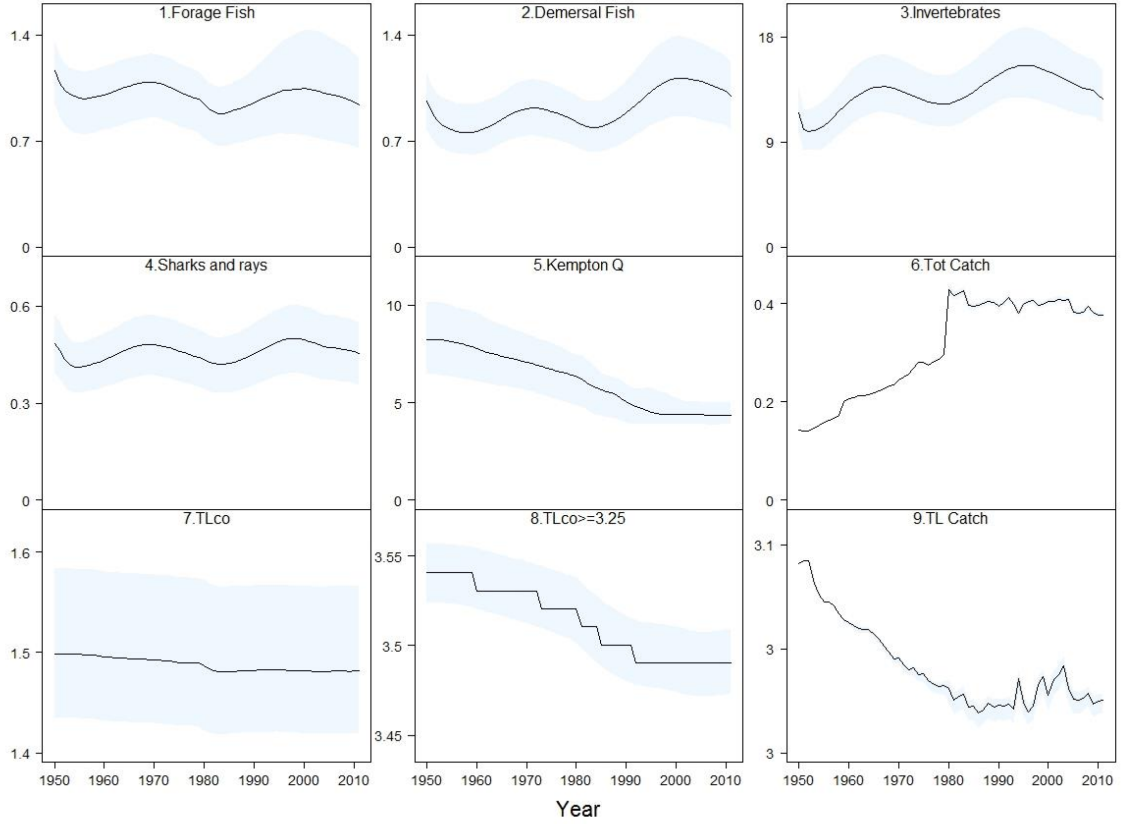

S13. Ecological indicators (1. Forage fish biomass (t·km^{-2}); 2. Demersal fish biomass (t·km^{-2}); 3. Invertebrates biomass (t·km^{-2}); 4. Sharks/rays and skate biomass (t·km^{-2}); 5. Kempton's index of biodiversity; 6. Tot Catch: Total Catch (t· km^{-2} ·year^{-1}); 7. mTLco: Mean trophic level of the community; 8. mTL>3.25: Mean trophic levels of groups having trophic level >3.25 (excluding marine mammals, sea turtles and seabirds); 9 mTLC: Mean trophic level of the catches) estimated from results of the Ecosim model for the period 1950-2011 for the Eastern Mediterranean Sea. Blue shadow represents the 95% percentile and 5% percentile obtained through the Monte Carlo routine.

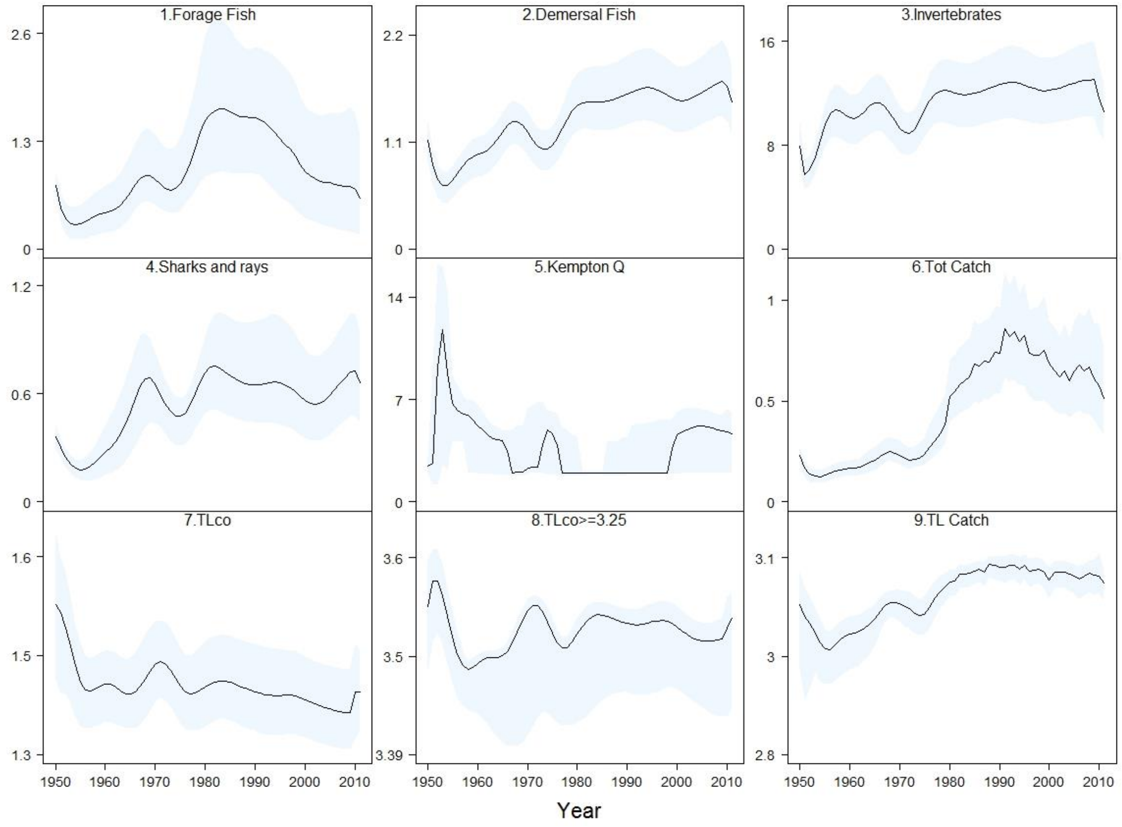

S14. Final vulnerabilities, obtained after the fitting procedure, for each functional groups of the sub-regions (W: Western; A: Adriatic; I: Ionian and E: Eastern) and of the whole Medierranean Sea. Top-down controls are given by vulnerabilities >3, bottom-up controls by vulnerabilities <2, while mixed effect interactions are given by vulnerabilities (v) 2 ≤ v ≤ 3.

#	Predator functional group	M	W	A	I	E
**1**	**Piscivourous Cetaceans**		1.0	1.0	2	1
**2**	**Other cetaceans**	2.0	1.0E+10	2.0	5.4	2.0
**3**	**Pinnipeds**		2.0	1.0	1.0	2.0
**4**	**Seabirds**		1.0E+10	1.0E+10	147.2	1.0
**5**	**Sea turtles**	2.0	2.0	1.0	2.0	1.0E+10
**6**	**Large Pelagics**	1.0	1.0	1.0	1.0	1.0E+10
**7**	**Medium Pelagics**		1.1	4.0	2.0	1.0
**8**	**European pilchards**		2.1	1.0	1.0	1.3
**9**	**European anchovies**		3.5	1.0	1.0	1.5
**10**	**Other Small Pelagics**		1.7	1.0	13.8	1.0
**11**	**Large Demersals**		4.1	1.0E+10	1.0	1.0E+10
**12**	**European Hake**		1.2	1.0	1.0	1.0
**13**	**Medium Demersals**		1.5	1.0	1.0	1.0
**14**	**Small Demersals**		3.0	1.0	1.9	3.4
**15**	**Deep-sea Fish**		1.0E+10	1.0	1.0	1.0
**16**	**Sharks**		1.8	1.0	1.9	1.0
**17**	**Rays and skates**		1.0E+10	1.0	1.0	5.5
**18**	**Benthopelagic Cephalopods**		1.0	1.0	1.1	1.0
**19**	**Benthic Cephalopods**		4.1	1.0	1.0	3.2
**20**	**Bivalves and gastropods**		1.0E+10	1.0	5.8	2.0
**21**	**Crustaceans**		1.0	1.0	2.4	5.7
**22**	**Jellyfish**		1.0E+10	1.0E+10	1.0E+10	1.0
**23**	**Benthos**		1.0	1.0	1.0	1.0
**24**	**Zooplankton**		1.0	1.0E+10	1.0E+10	1.0E+10

References

1 Piroddi, C., Coll, M., Steenbeek, J., Moy, D. M. & Christensen, V. Modelling the Mediterranean marine ecosystem as a whole: addressing the challenge of complexity. *Mar Ecol Prog Ser* **533**, 47-65 (2015).

2 Aguilar, A. Current status of Mediterranean monk seal (Monachus monachus) populations. *IUCN, Gland, Switzerland* (1998).

3 Sergeant, D., Ronald, K., Boulva, J. & Berkes, F. The recent status of Monachus monachus, the Mediterranean monk seal. *Biological Conservation* **14**, 259-287 (1978).

4 Panou, A., Jacobs, J. & Panos, D. The endangered Mediterranean monk seal Monachus monachus in the Ionian Sea, Greece. *Biological Conservation* **64**, 129-140 (1993).

5 Adamantopoulou, S. *et al.* Movements of Mediterranean monk seals (Monachus monachus) in the eastern Mediterranean Sea. *Aquatic Mammals* **37**, 256 (2011).

6 Antolović, J. *et al.* Protection of the Mediterranean Monk Seal (Monachus monachus) and its habitats. *Rapport Du 36e Congrés de la Commission Internationale pour l'Exploration Scientifique de la mer Méditerranée. Monte Carlo (Monaco)*, 36-230 (2001).

7 Notarbartolo di Sciara, G., Adamantopoulou, S., Androukaki, E., Dendrinos, P., Karamanlidis, A., Paravas, V., Kotomatas, S. . National strategy and action plan for the conservation of the Mediterranean monk seal in Greece, 2009 - 2015. Report on evaluating the past and structuring the future. (Hellenic Society for the Study and Protection of the Mediterranean monk seal (MOm), Athens, Greece, 2009).

8 Hellenic Society for the Study and Protection of the Mediterranean monk seal (MOm). Status of the Mediterranean monk seal *Monachus monachus* in *Greece*. 36 (Athens, Greece, 2009).

9 Mo, G., Agnesi, S., Di Nora, T. & Tunesi, L. Mediterranean monk seal sightings in Italy through interviews: Validating the information (1998–2006). *Rapport Commission International Mer Méditerranée* **38**, 542 (2007).

10 Gomerčić, T., Huber, D., Gomerčić, M. & Gomerčić, H. Presence of the Mediterranean monk seal (Monachus monachus) in the Croatian part of the Adriatic Sea. *Aquatic Mammals* **37**, 243-247 (2011).

11 Güçlüsoy, H., Kiraç, C., Veryeri, N. O. & Savas, Y. Status of the Mediterranean monk seal, Monachus monachus (Hermann, 1779) in the coastal waters of Turkey. *EU Journal of Fisheries & Aquatic Sciences* **21**, 201-210 (2004).

12 Gucu, A. C., Gucu, G. & Orek, H. Habitat use and preliminary demographic evaluation of the critically endangered Mediterranean monk seal (Monachus monachus) in the Cilician Basin (Eastern Mediterranean). *Biological Conservation* **116**, 417-431 (2004).

13 Scheinin, A. P., Goffman, O., Elasar, M., Perelberg, A. & Kerem, D. H. Mediterranean monk seal (Monachus monachus) resighted along the Israeli coastline after more than half a century. *Aquatic Mammals* **37**, 241 (2011).

14 Casale, P. & Margaritoulis, D. *Sea turtles in the Mediterranean: distribution, threats and conservation priorities*. (IUCN, 2010).

15 Margaritoulis, D. *et al.* Loggerhead turtles in the Mediterranean Sea: present knowledge and conservation perspectives. *Loggerhead Sea Turtles (editors: AB Bolten, BE Witherington). Smithsonian Institution Press, Washington DC* (2003).

16 Margaritoulis, D. & Rees, A. F. The loggerhead turtle, Caretta caretta, population nesting in Kyparissia Bay, Peloponnesus, Greece: results of beach surveys over seventeen seasons and determination of the core nesting habitat. *Zoology in the Middle East* **24**, 75-90 (2001).

17 Mingozzi, T. *et al.* in *Biodiversity and Conservation in Europe* 277-299 (Springer, 2008).

18 Lauriano, G., Panigada, S., Casale, P., Pierantonio, N. & Donovan, G. Aerial survey abundance estimates of the loggerhead sea turtle Caretta caretta in the Pelagos Sanctuary, northwestern Mediterranean Sea. *Mar Ecol Prog Ser* **437**, 291-302 (2011).
19 Kasparek, M., Godley, B. J. & Broderick, A. C. Nesting of the green turtle, Chelonia mydas, in the Mediterranean: a review of status and conservation needs. *Zoology in the Middle East* **24**, 45-74 (2001).
20 Kasparek, M. & Baran, İ. *Marine turtles, Turkey: Status survey 1988 and recommendations for conservation and management*. (World Wide Fund for Nature, 1989).
21 Groombridge, B. *Marine turtles in the Mediterranean: distribution, population status, conservation*. (Council of Europe, 1990).
22 Canbolat, A. F. A review of sea turtle nesting activity along the Mediterranean coast of Turkey. *Biological Conservation* **116**, 81-91 (2004).
23 Camiñas, J. A. Sea turtles of the Mediterranean Sea: population dynamics, sources of mortality and relative importance of fisheries impacts. *FAO fisheries report*, 27-84 (2004).
24 Broderick, A. C. & Godley, B. J. Population and nesting ecology of the green turtle, Chelonia mydas, and the loggerhead turtle, Caretta caretta, in northern Cyprus. *Zoology in the Middle East* **13**, 27-46 (1996).
25 Broderick, A. C., Glen, F., Godley, B. J. & Hays, G. C. Estimating the number of green and loggerhead turtles nesting annually in the Mediterranean. *Oryx* **36**, 227-235 (2002).
26 Bentivegna, F. *et al.* Loggerhead turtle (*Caretta caretta*) nests at high latitudes in Italy: a call for vigilance in the Western Mediterranean. *Chelonian Conservation and Biology* **9**, 283-289 (2010).
27 Ahrens, R. N. M. *A global analysis of apparent trends in abundance and recruitment of large tunas and billfishes inferred from Japanese longline catch and effort data* PhD thesis, University of British Columbia, (2010).
28 MEDIAS. Report of 5th meeting for MEDIterranean Acoustic Surveys in the framework of European Data Collection Framework (DCF) (Sliema, Malta, 2012).
29 MEDIAS. Report of 3rd meeting for MEDiterranean Acoustic Surveys (MEDIAS) in the framework of European Data Collection Framework. (Capo Granitola, Sicily, 2010).
30 Sinovčić, G., Zorica, B., Keč, V. Č. & Mustać, B. Inter-annual fluctuations of the population structure, condition, length-weight relationship and abundance of sardine, Sardina pilchardus (Walb., 1792), in the nursery and spawning ground (coastal and open sea waters) of the eastern Adriatic Sea (Croatia). *Acta Adriatica* **50**, 11-21 (2009).
31 Santojanni, A. *et al.* Stock assessment of sardine (Sardina pilchardus, Walb.) in the Adriatic Sea with an estimate of discards. *Scientia Marina* **69**, 603-617 (2005).
32 Bedairia, A. & Djebar, A. B. A preliminary analysis of the state of exploitation of the sardine, Sardina pilchardus (Walbaum, 1792), in the gulf of Annaba, East Algerian. *Animal biodiversity and conservation* **32**, 89-99 (2009).
33 Patti, B. *et al.* Interannual fluctuations in acoustic biomass estimates and in landings of small pelagic fish populations in relation to hydrology in the Strait of Sicily. *Chemistry and Ecology* **20**, 365-375 (2004).
34 García, A. & Giraldez, A. Small pelagic fish research in the Mediterranean by the Spanish Institute of Oceanography: available data series for a climatic analysis. (Fuengirola, Spain, 2012).

35 De Felice, A. Biomass evaluation of anchovy (E. encrasicolus), sardine (S. pilchardus) and sprat (S. sprattus) in the western Adriatic Sea by means of acoustics and preliminary analysis of possible relationships with environmental parameters. (Fuengirola, Spain, 2012).
36 STECF. Assessment of Mediterranean Sea stocks part I. (Luxembourg, 2010).
37 STECF. Assessment of Mediterranean Sea stocks part I (STECF 11-08). (Luxembourg, 2011).
38 STECF. Assessment of Mediterranean Sea stocks part I (STECF 12-19). (Luxembourg, 2012).
39 STECF. Assessment of Mediterranean Sea stocks part I (STECF 13-22). (Luxembourg, 2013).
40 STECF. Assessment of Mediterranean Sea stocks part II. (Luxembourg, 2010).
41 STECF. Assessment of Mediterranean Sea stocks part II (STECF-14-08). (Luxembourg, 2013).
42 STECF. Assessment of Mediterranean Sea stocks part II (STECF 11-14). (Luxembourg, 2011).
43 STECF. Assessment of Mediterranean Sea stocks part II (STECF 13-05). (Luxembourg, 2012).
44 Fiorentino, F. *et al.* A comparison between acoustic and bottom trawl estimates to reconstruct the biomass trends of sardine and anchovy in the Strait of Sicily (Central Mediterranean). *Fisheries Research* **147**, 290-295 (2013).
45 Jardim, E. *et al.* Stock assessment of Hellenic small pelagic stocks. EUR 27484. Luxembourg (Luxembourg): Publications Office of the European Union; 2015. JRC97817. (2015).
46 Wei, C.-L. *et al.* Global patterns and predictions of seafloor biomass using random forests. *PloS one* **5**, e15323 (2010).
47 Pauly, D. & Palomares, M. An empirical equation to predict annual increases in fishing efficiency. Fisheries Centre University of British Columbia Working Paper Series 07. (2010).
48 Damalas, D. *et al.* “Once upon a Time in the Mediterranean” long term trends of mediterranean fisheries resources based on fishers’ traditional ecological knowledge. *PloS one* **10**, e0119330 (2015).
49 EVOMED. The 20th Century evolution of Mediterranean exploited demersal resources under increasing fishing disturbance and environmental change. . (2011).
